# Climate warming and heatwaves accelerate global lake deoxygenation

**DOI:** 10.1126/sciadv.adt5369

**Published:** 2025-03-21

**Authors:** Yibo Zhang, Kun Shi, R. Iestyn Woolway, Xiwen Wang, Yunlin Zhang

**Affiliations:** ^1^ Key Laboratory of Lake and Watershed Science for Water Security, Nanjing Institute of Geography and Limnology, Chinese Academy of Sciences, Nanjing, China.; ^2^ University of Chinese Academy of Sciences, Beijing, China.; ^3^ School of Ocean Sciences, Bangor University, Menai Bridge, Anglesey, Wales.; ^4^ School of Geography and Ocean Science, Nanjing University, Nanjing, China.; ^5^ University of Chinese Academy of Sciences, Nanjing (UCASNJ), Nanjing, China.

## Abstract

A widespread decline in dissolved oxygen (DO) has been observed in rivers, temperate lakes, and oceans, yet the impacts of climatic warming on global lake deoxygenation remain unclear. Here, we train data-driven models using climatic data, satellite images, and geographic factors to reconstruct surface DO and quantify the climatic contribution to DO variations in 15,535 lakes from 2003 to 2023. Our analysis indicates a continuous deoxygenation in 83% of the studied lakes. The mean deoxygenation rate in global lakes (−0.049 milligrams per liter per decade) is faster than that observed in the oceans and in rivers. By decreasing solubility, climatic warming contributes 55% of global lake deoxygenation. Meanwhile, heatwaves exert rapid influences on DO decline, resulting in a 7.7% deoxygenation compared to that observed under climatological mean temperatures. By the end of the century, global lake DO is projected to decrease by 0.41 milligrams per liter (4.3%) under SSP2-4.5 and 0.86 milligrams per liter (8.8%) under SSP5-8.5 scenarios.

## Teaser

Both long-term climate change and short-term heatwaves have a profound impact on dissolved oxygen levels in global lakes.

## INTRODUCTION

The persistent decline in dissolved oxygen (DO) concentrations, observed across diverse aquatic ecosystems since the mid-20th century, has prompted substantial concern. Global observations demonstrate a widespread decline (2%) in DO concentrations across the open oceans, leading to the proliferation of “dead zones,” areas characterized by very low DO (*1*). Similarly, diminishing oxygen levels have been observed in coastal regions (−0.15 mg/liter per decade) (*2*) and in rivers (−0.038 mg/liter per decade) (*3*), with hundreds of sites reporting hypoxic conditions characterized by DO concentrations of ≤2 mg/liter in recent decades (*4*). Recent investigations have further suggested a decline in DO concentrations in temperate lakes, showing reductions of 0.45 mg/liter (5.5%) in surface waters and 0.42 mg/liter (18.6%) in deep waters from 1980 to 2017 (*5*). These declines in DO can critically disrupt the delicate balance of an ecosystem, particularly since DO serves as a pivotal factor in driving biological and biogeochemical processes (*5*, *6*). Adequate oxygen levels are critical for sustaining aerobic life and fostering robust biological communities (*7*, *8*). A decrease in DO concentrations results in substantial consequences, including reduced nitrogen fixation, increased emissions of N_2_O—a potent greenhouse gas (*4*), limitations on habitat suitability and productivity for oxygen-demanding organisms (*9*), as well as having adverse impacts on food security, livelihoods (*4*), and coastal economies (*1*, *7*, *9*, *10*).

The primary driver of surface deoxygenation in lakes, as well as in the oceans and in rivers, is the global increase in water temperature (*1*, *4*, *5*). This phenomenon predominantly arises from diminishing solubility, which in the oceans is estimated to account for approximately 50% of the total oxygen loss in the upper 1000 m (*1*). In lakes and oceans, surface warming also impedes vertical oxygen transport from the surface, where it is typically highest, into deeper waters by strengthening and prolonging thermal stratification (*4*, *11*). This disruption in vertical mixing (*11*) can lead to a critical depletion of DO in bottom waters (*1*, *12*). Furthermore, elevated lake water temperature can potentially influence DO concentrations by stimulating the growth of aquatic vegetation and phytoplankton (*4*, *13*), consequently enhancing both oxygen consumption and production rates (*5*, *14*). When oxygen consumption through respiration exceeds the rate of oxygen supplied by photosynthesis over extended periods, DO concentrations naturally decrease and may become depleted (*4*). This phenomenon is widespread across various aquatic systems (*15*, *16*). Conversely, in situations where oxygen supply from photosynthesis exceeds DO consumption, DO concentrations will increase (*5*) and can lead to DO supersaturation as observed in numerous systems, including eutrophic lakes and those with high aquatic vegetation (*4*, *5*, *13*, *17*).

Traditionally, studies investigating the impact of climate warming on DO have primarily centered on oceanic, coastal, and riverine systems (*1*, *4*, *18*, *19*). However, recent research has increasingly focused on understanding the mechanisms driving changes in DO concentration across large spatial scales, notable in temperate lakes (*5*, *20*). Nevertheless, it is essential to recognize the nonlinear relationship between temperature and oxygen solubility, which make explanation of warming impact on DO unreliable from one region to another. Consequently, conclusions drawn from studies on temperate lakes may not be transferrable to lakes in other regions, such as the tropics. In these areas, higher surface water temperatures result in a reduced sensitivity of DO concentrations to oxygen solubility. Moreover, current research primarily offers qualitative insights into the relationship between long-term climate change and DO concentrations. However, there is a notable gap in our understanding of the quantitative effects of both continuous climate warming and the increased occurrence and severity of short-term heatwave events (*21*, *22*) on global DO dynamics. Investigations into specific lakes have demonstrated that heatwaves can trigger rapid and substantial fluctuations in DO concentrations over short periods (*23*, *24*), emphasizing the crucial role of heatwaves in influencing the oxygen dynamics of aquatic ecosystems, but a global analysis is now lacking.

In this study, we used model-derived DO estimates for 15,535 lakes to investigate the extent to which (i) long-term climate warming and (ii) short-term heatwaves contribute to surface DO dynamics on a global scale. To achieve this objective, we use a machine learning approach (figs. S1 and S2), which incorporates commonly recognized climatic parameters, namely, air temperature, solar and thermal radiation, wind speed, atmospheric pressure, and precipitation. In addition, geographic factors such as elevation and latitude are incorporated, enhancing the model’s ability to capture spatial variations in environmental conditions. Moreover, two water quality indices, namely, the floating algae index (FAI) (*25*) and hue angle (*26*), are also integrated (fig. S1). The FAI is defined as the difference between the reflectance in the near-infrared band and the linear baseline established by the red and short-wave infrared bands. It provides insights into phytoplankton biomass, where elevated FAI values indicate dense aggregations of surface phytoplankton (*27*); while hue angle stands as a straightforward metric for assessing trophic status (*28*). Subsequently, the developed machine learning model is used to retrospectively reconstruct DO records spanning various temporal scales (annual and monthly) from 2003 to 2023. Furthermore, we examine the interaction between DO saturation and eutrophication and explore the impact of climate warming and eutrophication on DO concentrations using a structural equation modeling (SEM) approach. In addition, historical trends in heatwaves are scrutinized, and their impact on DO concentrations is quantitatively assessed. Last, prospective DO trends and the potential emergence of stressed condition—defined as DO concentrations below 6.0 mg/liter, a threshold at which fish growth and food consumption notably decline in freshwater ecosystems (*29*, *30*)—are projected under two Shared Socioeconomic Pathways (SSP) scenarios: SSP2-4.5 and SSP5-8.5.

## RESULTS

### Spatiotemporal variations in DO of global lakes

**Fig. 1.**
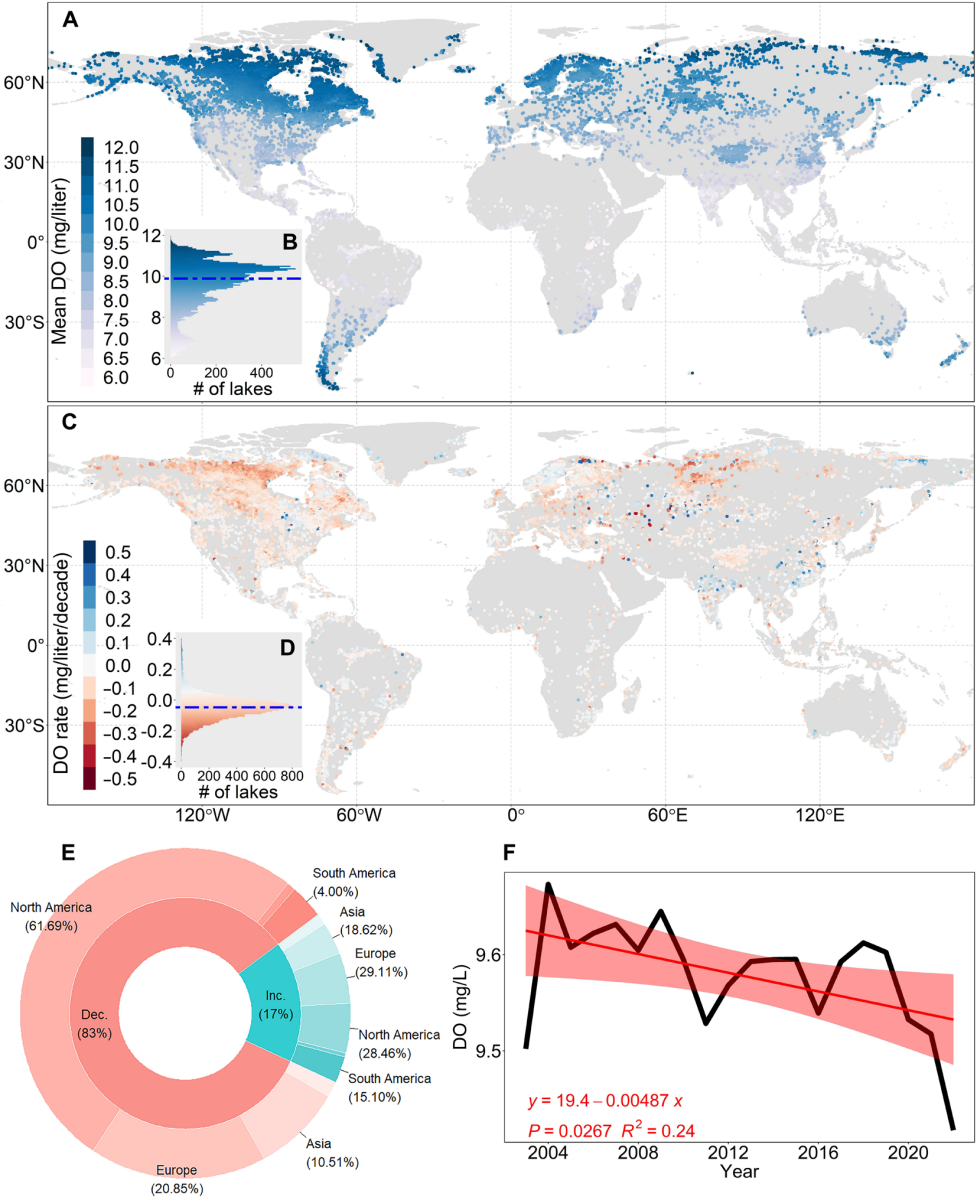
Spatiotemporal variations in DO concentrations for global lakes with surface areas ≥ 10 km^2^. (**A**) The spatial distribution of mean DO. (**B**) Statistical histograms illustrating the mean DO, with horizontal dashed lines representing median values. (**C**) The spatial distribution of the change rate of DO. (**D**) Statistical histograms illustrating the change rate of DO, with horizontal dashed lines representing median values. (**E**) Statistical information of the DO rate across two categories: increase (Inc.) and decrease (Dec.), for each continent. (**F**) Decadal trends spanning from 2003 to 2023 for the mean DO on a global scale, with the red color representing the 95% confidence interval.

After conducting model comparison (see the “Model development” section in Materials and Methods), the random forest (RF) model, which exhibited superior accuracy in simulating DO concentrations, was selected to estimate DO concentrations in global lakes. The annual mean surface DO concentration in 15,535 globally modeled lakes from 2003 to 2023 was 9.76 ± 1.32 mg/liter (mean ± SD) (Fig. 1, A and B). Lakes in North America and Europe exhibited significantly higher surface DO concentrations compared to other continents [analysis of variance (ANOVA) test, *P* < 0.001], with multiyear averages of 10.05 ± 0.90 and 10.00 ± 0.74 mg/liter, respectively (Fig. 1A and fig. S3A). The spatial distribution and regional dynamics of surface DO are presented in Fig. 1C and fig. S3B. Globally, lakes exhibit an overall decreasing trend in surface DO with a mean rate of −0.048 mg/liter per decade during the study period (Fig. 1D). Specifically, lakes situated across six continents have experienced declines over the past two decades by −0.03 mg/liter in Africa, −0.024 mg/liter in Asia, −0.074 mg/liter in Europe, −0.13 mg/liter in North America, −0.052 mg/liter in Oceania, and −0.012 mg/liter in South America, respectively (Fig. 1C and fig. S3B). In contrast, lakes with increasing DO concentrations appear to be predominantly located in tropical regions (Fig. 1A). Globally, 17% of the studied lakes experienced an increase in surface DO concentrations, with an average rate of 0.064 mg/liter per decade; while 83% experienced a decrease in surface DO concentration, averaging −0.079 mg/liter per decade (Fig. 1E). Overall, lake surface DO decreased globally by −0.10 mg/liter during the past two decades (*t* test, slope = −0.049 mg/liter per decade, *P* = 0.026) (Fig. 1F). The rates of change in lake surface DO is faster than those observed in the oceans (−0.022 mg/liter per decade) (*1*) and in rivers (−0.038 mg/liter per decade) over a similar time period (*3*).

Seasonal surface DO fluctuations (∆_DO_) of the studied lakes show greater variability compared to their interannual variations. The range of ∆_DO_ varies from 0.31 to 6.17 mg/liter, with a mean value of 2.75 ± 1.01 mg/liter (fig. S4, A and B). Seasonal surface DO fluctuations are greater in the Northern Hemisphere (mean ∆_DO_ = 2.85 ± 0.96 mg/liter) and smaller in the Southern Hemisphere (mean ∆_DO_ = 1.64 ± 0.86 mg/liter) (fig. S4A). Seasonal variations in DO concentrations display divergent patterns between the Southern Hemisphere and the Northern Hemisphere (fig. S4, A and B). In the Southern Hemisphere, mean DO levels peak around August and reach their minimum in January (fig. S4C). Conversely, in the Northern Hemisphere, surface DO levels peak around March and are lowest around July (fig. S4D). Furthermore, the monthly averaged DO in the Southern and Northern Hemispheres exhibits contrasting trends, with a convex pattern in the Southern Hemisphere regions (fig. S4E) and a combination of both convex and concave patterns in the Northern Hemisphere regions (fig. S4F).

### Impact of climate warming on global lake deoxygenation

**Fig. 2.**
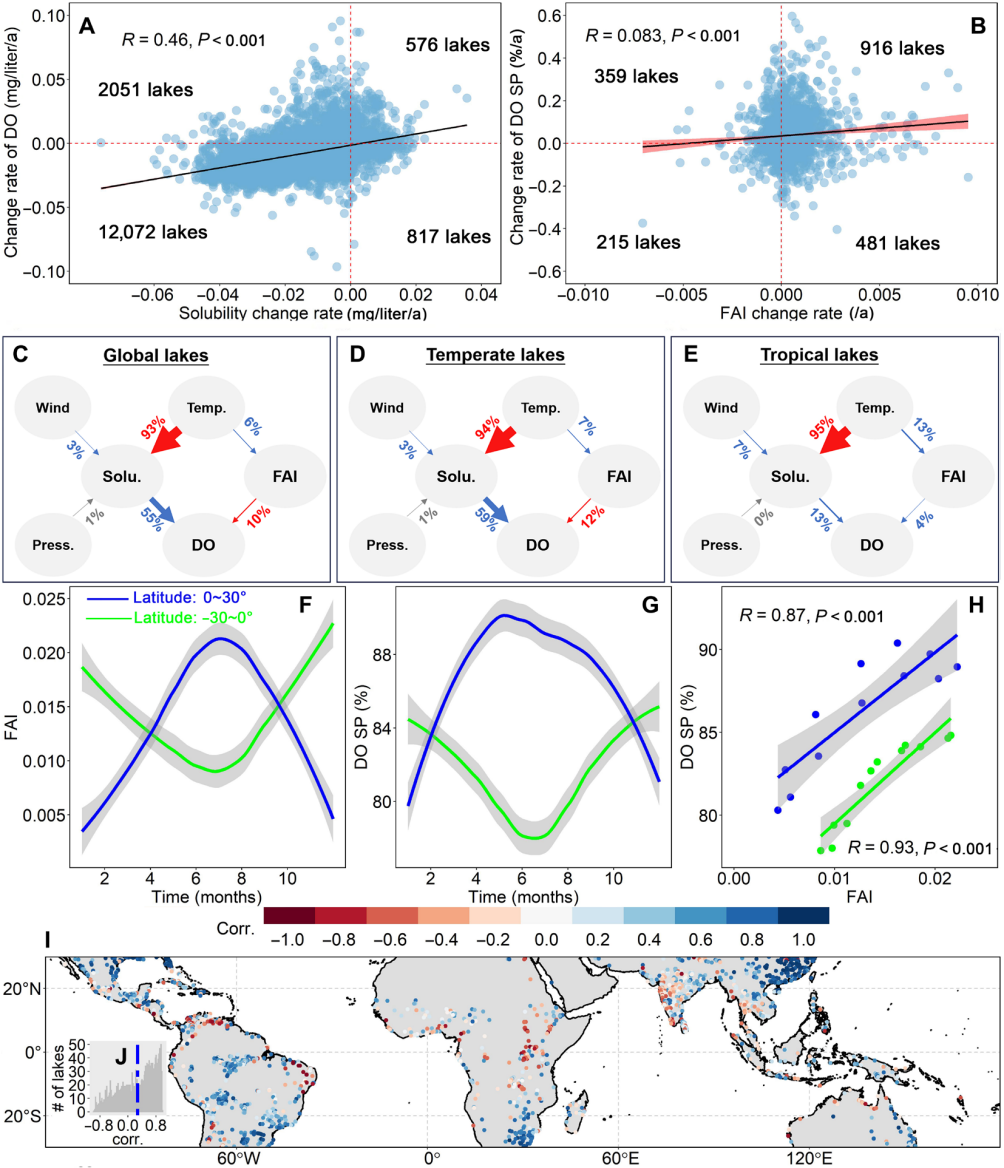
Impact of climate warming on global lake deoxygenation. Solubility and FAI effects on DO level over time. (**A**) Estimated DO concentration rate versus solubility rate for global lakes (*n* = 15,533). (**B**) Estimated DO SP rate versus FAI rate for tropical lakes (*n* = 1971). (**C** to **E**) Mean contributions of long-term climate changes (temperature, wind speed, and atmospheric pressure) to solubility variation, as well as the long-term changes in solubility and FAI to DO variation for global lakes (C), temperate lakes (D), and tropical lakes (E). Red color indicates a negative impact, while blue color indicates a positive impact, and gray color signifies little to no effect. (**F**) Seasonal trend of FAI for lakes within latitudes of 0° ~ 30° (blue) and −30° ~ 0° (green). (**G**) Seasonal trend of DO SP for lakes within latitudes of 0° ~ 30° (blue) and −30° ~ 0° (green). (**H**) Linear fitting between monthly DO SP and FAI for lakes within latitudes of 0° ~ 30° (blue) and −30° ~ 0° (green). (**I**) Spatial distribution of correlation between seasonal DO SP and FAI for lakes within latitudes of −30° ~ 30°. (**J**) Statistical histograms illustrating the correlation, with a blue vertical line representing the mean value.

The rate of change in surface DO concentration is strongly correlated with the rate of change in its solubility (Supplementary Text 1) (*R* = 0.46, *P* < 0.001) in global lakes (Fig. 2A), with a substantial number of lakes experiencing declines in both DO solubility (DOsol) (fig. S5) and DO concentrations (*n* = 12,072) (Figs. 1 and 2A). Departures of DO concentrations from 100% saturation (DO SP) (Supplementary Text 1) capture the influence of photosynthesis, respiration, and other chemical and biogeochemical processes that produce or consume oxygen (*3*). During the past two decades, global DO SP has increased by 0.88% (fig. S6) despite DOsol decreasing by −0.24 mg/liter (Supplementary Text 1 and fig. S5). The observed increase in global DO SP could be attributed to several factors, including the rising occurrence and intensity of phytoplankton blooms (*27*, *31*, *32*). Analysis of the interaction between DO SP and eutrophication (measured as FAI) among tropical lakes indicates that DO SP generally increases with increasing FAI, with a substantial number of lakes experiencing increases in both DO SP and FAI (*n* = 916) (Fig. 2B).

Our investigation quantified the contributions of long-term climate change (temperature, wind speed, and atmospheric pressure) to variations in solubility, as well as the long-term effects of solubility and eutrophication on DO variability lakes worldwide (refer to Materials and Methods). The results indicate that temperature generally shows a notable negative contribution to solubility in nearly all lakes, while other pathways exhibit both positive and negative contributions (fig. S7). On the basis of the SEM results of each lake, we calculated the mean contributions of different pathways. Our analysis suggests that atmospheric pressure has little to no effect on solubility, with a mean contribution close to 0 for both temperate and tropical lakes. Wind speed shows a small positive contribution, with a mean contribution of 3% for temperate and 7% for tropical lakes (Fig. 2, C to E). The decrease in DOsol is estimated to account for 55% of total global surface oxygen loss, which is slightly higher than that in the oceans (~50%) (*1*, *4*). However, the DOsol decrease does not fully explain global lake oxygen decline. The negative contribution of FAI to DO variation (fig. S1) suggests that oxygen consumption surpasses oxygen supply due to an increase in eutrophication, resulting in a net decrease in DO concentration. Specifically, increasing eutrophication is estimated to account for 10% of total global surface oxygen loss (Fig. 2C). The mechanisms driving DO change differ between temperate and tropical lakes. In temperate lakes, the positive contribution from solubility is 59%, and the negative contribution from FAI increases slightly to 12% (Fig. 2D). In tropical lakes, the contribution from solubility is notably decreasing to 13%, while the FAI impact changes from negative to positive (4%) (Fig. 2E). The observed increase in FAI may help explain the rise in DO concentrations in tropical lakes (Fig. 1A). This suggests that oxygen supply of photosynthesis by eutrophication outweighs DO consumption, consistent with recent studies identifying photosynthesis of cyanobacteria blooms as the primary factor leading to DO supersaturation in eutrophic lakes when water temperature exceeded around 25°C (*5*). This result suggests that the sensitivity of DO to temperature decreases as temperatures increase in tropical lakes. Increasing temperature might lead to greater eutrophication, which in turn stimulates increased photosynthetic activity among algae. This not only counteracts the decline of DO concentration from changing solubility but paradoxically results in a net increase in DO concentration. In other words, eutrophication, as a primary control, increases water DO concentration in some tropical lakes.

The seasonal temperature differences between the Northern and Southern Hemispheres primarily drive divergent patterns of seasonal variation in surface DO concentrations (fig. S4, A and B), as the seasonal patterns of DO in both hemispheres are highly opposite to the seasonal variations in temperature observed in previous studies (*33*, *34*). In the Southern Hemisphere, the highest surface DO corresponds to the lowest temperatures around August, while the lowest DO occurs during the highest temperatures around January (fig. S4C). In contrast, in the Northern Hemisphere, the highest surface DO levels correspond to the lowest temperatures around March, and the lowest DO coincides with the highest temperatures around July (fig. S4D). Further analysis of the relationship between seasonal DO SP and trophic state revealed consistent trends in both the Northern and Southern tropics (Fig. 2, F and G). A robust positive correlation between DO SP and FAI was observed, with correlation coefficients of 0.87 for lakes in the Northern tropics and 0.93 for those in the Southern tropics (Fig. 2H). Individual lake assessments showed that 70% exhibited a positive correlation between seasonal DO SP and FAI (Fig. 2, I and J). Thus, we assert that seasonal fluctuations in eutrophication have a more substantial impact on DO concentration than annual fluctuations.

### Impact of heatwave incidence on global lake deoxygenation

On the basis of ERA5 reanalysis data, we identified atmospheric heatwave events over global lakes during the period of 2003–2023 (the definition of heatwave events can be found in Materials and Methods). The average duration of heatwaves over global lakes was 15 days per year (fig. S8). Specifically, the duration of heatwaves is longer near the equator compared to high-latitude regions (fig. S8A). The spatial distribution of the change rate of heatwave duration is outlined in fig. S8C. Globally, 85% of the studied lakes have experienced a gradual increase in the number of heatwave days per year. Specifically, the number of heatwave days has increased across all six continents over the past two decades, with an increase rate of 1.2 days/year in Africa, 0.7 days/year in Asia, 0.6 days/year in Europe, 0.5 days/year in North America, 1.4 days/year in Oceania, and 0.6 days/year in South America, respectively (fig. S9).

**Fig. 3.**
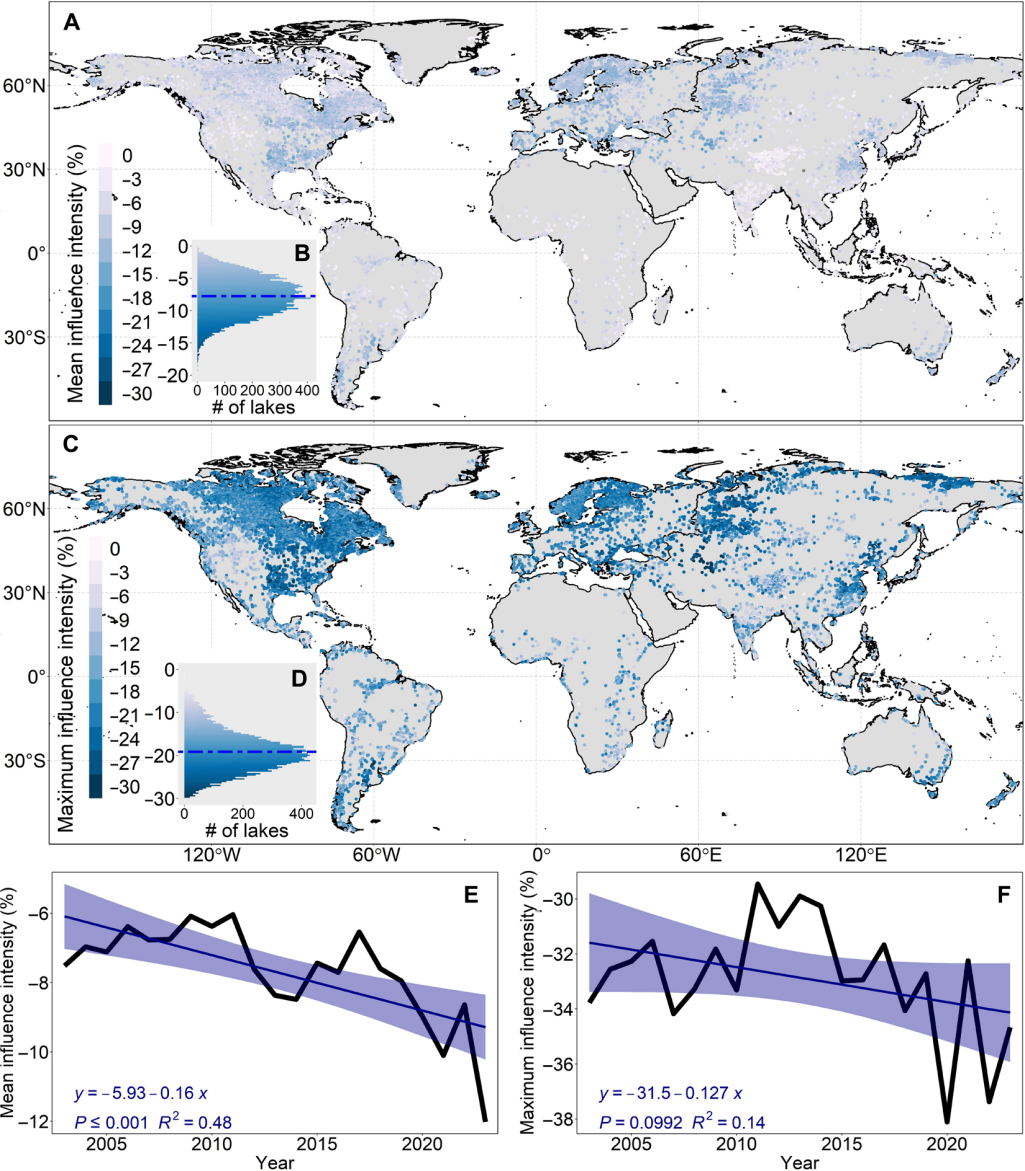
Impact of heatwave incidence on global lake deoxygenation. The influence intensity represents the mean percentage relative difference between the two calculated DO values driven by actual temperature and the climatological mean temperature within the period of 1970–1999. The mean influence intensity reflects the average influence intensity during heatwave events, while the maximum influence intensity indicates the maximum intensity observed during heatwave events. (**A**) Spatial distribution of the mean influence intensity for global lakes with areas of ≥10 km^2^. (**B**) Statistical histogram illustrating the mean influence intensity of heatwaves on DO concentration, with a horizontal dashed line representing the mean value. (**C**) Spatial distribution of the maximum influence intensity for global lakes with areas of ≥10 km^2^. (**D**) Statistical histogram illustrating the maximum influence intensity of heatwaves on DO concentration, with a horizontal dashed line representing the mean value. (**E**) Decadal trends of mean influence intensity from 2003 to 2023 at the global scale, with blue shading representing the 95% confidence interval. (**F**) Decadal trends of maximum influence intensity from 2003 to 2023 at the global scale, with blue shading representing the 95% confidence interval.

Heatwaves can result in a reduction in DO solubility and lead to rapid and substantial fluctuations in DO concentration over short durations. Our analysis demonstrates that heatwaves can negatively affect DO concentrations in lakes worldwide (Fig. 3, A and B). The intensity of heatwaves on DO concentration, represented by the mean percentage relative difference between DO values driven by actual temperature and climatological mean temperature, shows a mean decrease of 7.7%, with a maximum decrease of 19.0% in DO concentration during heatwave events (Fig. 3, B and D). The impact of heatwaves on DO concentrations varies across spatial scales (Fig. 3). Specifically, European lakes exhibit notably higher absolute values of mean and maximum intensities in comparison to other continents, with multiyear averages of 9.7 ± 2.7% and 21.2 ± 4.1%, respectively (fig. S10, A and B). This observation may be attributed to the nonlinear correlation between temperature and DO solubility, particularly emphasizing the heightened solubility variations evident in high-latitude regions under equivalent temperature fluctuations. The mean influence intensity exhibited a notable increasing trend in absolute value, increasing from 7.5% in 2003 to 12% in 2023 (Fig. 3E). Specifically, mean influence intensity has increased across all six continents, by 1.9% in Africa, 0.8% in Asia, 1.3% in Europe, 2.0% in North America, 0.8% in Oceania, and 0.3% in South America, respectively, over the past two decades (fig. S11). The absolute value of maximum influence intensity also exhibited an increasing but insignificant trend (*P* = 0.1; Fig. 3F). Specifically, the absolute value of the maximum influence intensity has increased across five continents (Africa, Asia, Europe, North America, and Oceania) (fig. S12).

We analyzed two DO metrics under two scenarios: (i) influenced by temperature variations, including extreme heatwaves, and (ii) excluding heatwave effects. The relative difference, normalized by DO values under actual temperatures, quantifies the impact of heatwaves on long-term lake deoxygenation (see Materials and Methods). Globally, heatwaves have a minimal effect, contributing a mean impact of −0.9% (range: −3 to 2%; fig. S13, A and B). Negative impacts were observed in 92% of lakes, particularly in high-latitude regions, while 8% of lakes, mainly in tropical areas, experienced positive effects. Slight differences in histogram distributions of mean DO and DO rate between scenarios (fig. S13, C and D) further confirm the limited influence of heatwaves on long-term deoxygenation.

### Future change in DO and the proliferation of stressed lakes

**Fig. 4.**
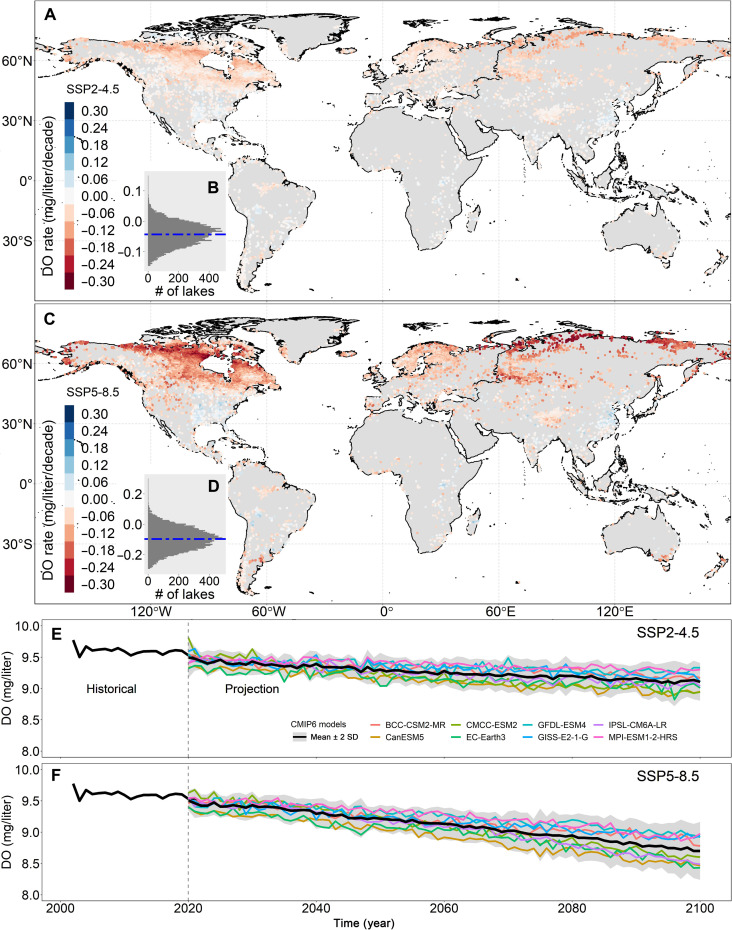
Projected changes in DO concentrations under SSP2-4.5 and SSP5-8.5 scenarios. (**A**) Spatial distribution of the projected DO rate under the SSP2-4.5 scenario. (**B**) Statistical histogram illustrating the DO rate under the SSP2-4.5 scenario, with a horizontal dashed line representing the mean value. (**C**) Spatial distribution of the projected DO rate under the SSP5-8.5 scenario. (**D**) Statistical histogram illustrating the DO rate under the SSP5-8.5 scenario, with a horizontal dashed line representing the mean value. (**E**) Projected DO concentrations under the SSP2-4.5 scenario. (**F**) Projected DO concentrations under the SSP5-8.5 scenario. Thick black lines are mean values for the historical period and projected future over global lakes. In the projected future, colored lines are the means of global lakes for each of the eight CMIP6 models, with gray shading indicating 2 SDs of the eight CMIP6 models.

We used the developed RF model to project future changes in DO. The model primarily uses daily climate variables, including solar radiation, thermal radiation, air temperature, air pressure, precipitation, and wind speed, as inputs. In addition, static variables such as elevation and absolute latitude, along with water environment factors like FAI and hue angle, were also included. The spatial patterns of projected DO under SSP2-4.5 and SSP5-8.5 scenarios exhibit similarities, yet they differ in terms of their magnitude of change (Fig. 4). Under SSP2-4.5, global DO is projected to decrease at a rate of −0.043 mg/liter per decade (Fig. 4, A and B). Deoxygenation is projected to occur more rapidly in Europe (−0.055 ± 0.032 mg/liter per decade) and North America (−0.048 ± 0.038 mg/liter per decade) compared to Africa (−0.017 ± 0.027 mg/liter per decade), Asia (−0.014 ± 0.032 mg/liter per decade), Oceania (−0.021 ± 0.031 mg/liter per decade), and South America (−0.014 ± 0.025 mg/liter per decade) (fig. S14). Under SSP5-8.5, projected DO rates (−0.097 mg/liter per decade) are approximately 2.3 times higher than those in SSP2-4.5. Our long-term projections suggest that the global mean DO will decrease to 9.11 ± 1.07 and 8.70 ± 0.89 mg/liter by 2100 under SSP2-4.5 and SSP5-8.5, respectively (Fig. 4, E and F).

Using our future projections, we investigated changes in the occurrence of stressed lakes, which we define as instances where DO drops below 6.0 mg/liter (refer to Materials and Methods). The projected stress frequency and stressed lakes were detected and presented in fig. S15. We predict that stressed lakes will primarily be in tropical regions (fig. S15, A to D). There are 238 (SSP2-4.5 scenario) and 279 (SSP5-8.5 scenario) lakes that are projected to experience stress conditions in the future. Although the numbers of low-frequency (<25%) lakes in the SSP2-4.5 scenario is shifting toward moderate frequency (25 to 75%) lakes in SSP5-8.5 scenario (fig. S15, B and D), the average frequency has not notably increased (32.09% in SSP2-4.5 scenario and 32.10% in SSP5-8.5 scenario, respectively). This is mainly due to an additional 41 lakes from SSP2-4.5 to SSP5-8.5 that experience stress conditions, which has lowered the average frequency. The number of stressed lakes generally exhibits an upward trend (fig. S15, E and F). In the historical records, an average of 53 lakes has been characterized by stress conditions. The number of stressed lake is projected to increase at a rate of 3.70 per decade under SSP2-4.5 and 7.03 per decade under SSP5-8.5. By 2100, the projected number of stress lakes is expected to reach 90 under SSP2-4.5 and 110 under SSP5-8.5, representing approximately 1.70 times and 2.08 times the historical figures (fig. S15, E and F).

## DISCUSSION

Despite the wide range of lake characteristics investigated in this study, the overall trend and mechanisms of DO declines are evident. The primary factor is the decreasing solubility due to warming, which accounts for 55% of surface oxygen loss in global lakes. Eutrophication, another contributor, is responsible for 10% of surface oxygen loss (Fig. 2). Eutrophication occurs when nutrients (primarily N and P) from human waste, agriculture, and atmospheric sedimentation stimulate the growth of algae and increase algal biomass (*4*, *35*). Although in some tropical regions, the oxygen supplied by photosynthesis due to eutrophication outweighs oxygen consumption, leading to an increase in final DO concentrations when the water temperature exceeds the balance point, where photosynthetic oxygen production equals respiratory oxygen consumption (*5*, *36*, *37*), elevated eutrophication levels intensify oxygen-consuming biological activities, such as algal respiration and microbial decomposition, thereby exacerbating deoxygenation in most regions globally (*3*, *4*). Despite active management efforts in many lakes (*38*–*40*) to mitigate phytoplankton growth by reducing nutrient loads, particularly in Europe and North America, success has been limited, and global algal blooms continue to proliferate in scale and magnitude (*32*, *41*, *42*). Although already rapid, future DO losses may accelerate, owing to continued decreases in solubility resulting from global warming. Projections suggest that global air temperatures are likely to rise to 1.5° and 3.5°C above preindustrial levels by 2100 under SSP2-4.5 and SSP5-8.5, respectively (*43*). As temperatures continue to increase, surface DO is projected to decrease by 0.34 and 0.76 mg/liter by 2100 under SSP2-4.5 and SSP5-8.5, respectively. In addition, as the frequency and impact intensity of heatwaves increase (figs. S9, S11, and S12), the future impact of heatwaves on lake deoxygenation may intensify, particularly in North America and Europe (Fig. 3). Our findings, therefore, suggest more rigorous efforts in the future to mitigate the impacts of climate warming, particularly the adverse effects of extreme heatwaves on DO deoxygenation in lakes worldwide.

Lakes in tropical regions demand attention due to the prevalence of stressed lakes (fig. S16). For instance, our predictions for Lake Victoria (one of the world’s largest lakes) (*44*) indicate a prolonged period of low-oxygen levels. In line with research by Ngupula *et al.* (*45*), offshore surface water exhibits lower DO levels compared to inshore water and often experiences stress conditions with DO levels below 6 mg/liter. Our synchronized predictions reveal that the stressed area expanded to a maximum of 28,760 km^2^ in 2007, encompassing 43% of the total lake surface (fig. S16). A further decline in oxygen levels could have substantial impacts on biological and biogeochemical cycling (*4*). Low DO levels affect biological processes by diminishing survival and growth and altering the behavior of individual organisms. Repeated exposure to low oxygen may lead to the loss of sensitive species, the death of aquatic organisms, and the collapse of commercial fisheries (*4*, *7*, *9*, *30*, *46*). Studies have demonstrated that nearly all fish cannot survive when DO levels decrease below ~3 mg/liter (*30*).

In polymictic lakes, a decrease in surface DO concentrations will also lead to a corresponding reduction in bottom-water DO, subsequently affecting methane (a potent greenhouse gas) production in anoxic sediments (*4*, *8*, *47*). In addition, at the oxic-suboxic boundaries of low-oxygenated waters, production of N_2_O, another greenhouse gas, is enhanced through denitrification and nitrification (*8*, *48*), potentially amplifying global warming (*49*). When oxygen levels are extremely low, anaerobic remineralization of organic matter through denitrification and anammox leads to the loss of bioavailable nitrogen through the generation of denitrification gas (N_2_) (*50*). The supply of phosphorus and iron released from the sediments is generally enhanced under anoxic conditions (*4*, *51*, *52*). These nutrients have the potential to stimulate algal growth, increase algal biomass, and consequently increase DO consumption, potentially leading to further release of phosphorus and iron from the sediment (*4*, *53*). Urgent mitigation measures are imperative given the current stress on DO levels and the anticipated trends in tropical lakes, alongside the looming threat of oxygen decline in lake ecosystems.

## MATERIALS AND METHODS

### In situ DO data

The in situ DO dataset used in this study was sourced from five distinct repositories: the lake surveys conducted within the Basin of the Yangtze River during the periods of 2007–2009 and 2018–2019, the regular assessments conducted in Lake Taihu spanning from 2002 to 2019, surveys carried out on lakes situated within the Qinghai-Tibetan Plateau from 2018 to 2021, the American National Aquatic Resource Surveys dataset from 2007 (accessible at https://epa.gov), and the Global Freshwater Quality dataset (accessible at https://gemstat.bafg.de/). The DO measurements were obtained in situ using calibrated optical DO meters deployed in the field (*54*). The DO observations from the mixed layer or deep water (depths, >1 m) were excluded, leaving only the surface-level observations (depths, ≤1 m) (*54*) for model construction.

### MODIS data processing

This study leveraged daily Moderate Resolution Imaging Spectroradiometer (MODIS) Aqua surface reflectance products (MODIS/061/MYD09GA) spanning the temporal domain from 2003 to 2023. The MODIS Aqua surface reflectance products have been shown to be useful for monitoring water quality in many lakes (*55*–*57*). Each acquired image was converted to water leaving reflectance by dividing π (3.14), followed by rigorous quality control procedures. These procedures involved the elimination of erroneous and cloud-contaminated pixels, as well as identification of potential artifacts such as atmospheric correction failures, using quality control flags (*55*). The optical depth (OD) was computed using bathymetric data (*58*) and inherent optical properties derived from the Quasi-Analytical Algorithm (QAA) (*59*). A criterion was then applied to mask optically shallow pixels, defined as those with OD values below 20 (*60*). Access to satellite scenes encompassing the study lakes was facilitated through the utilization of Google Earth Engine (GEE) (*61*), a cloud-based platform tailored for extensive geospatial analysis on a global scale.

### Lake water identification

Recognizing the temporal variability of water bodies, fixed lake boundaries were deemed inadequate, potentially encompassing nonwater areas such as inundation zones (*55*). To address this, a dynamic approach using the normalized difference water index (NDWI) and histogram splitting threshold for each lake, as per the methodology outlined by Hou *et al.* (*55*), was implemented to delineate water bodies in clear images. Permanent water bodies, defined as those with a >70% occurrence frequency (*62*), were identified and further refined through clipping and screening against a database of 16,689 lake polygons with surface areas exceeding 10 km^2^ sourced from HydroLAKES (*63*). To mitigate land adjacency effects, water bodies were eroded inward by one pixel (*55*), and lakes composed of fewer than four pixels were excluded (*64*). Last, 15,535 lakes were observed, on average, 28.5 times per year during the MODIS periods spanning 2003–2023 (fig. S17).

### Model input features

We incorporated three types of data for DO retrieval: (1) climate factors, (2) water quality factors, and (3) geographic factors.

1) Climate factors encompass solar radiation, thermal radiation, air temperature, air pressure, precipitation, and wind speed components *u* and *v*. These data are sourced from the European Centre for Medium-Range Weather Forecasts fifth-generation global atmospheric reanalysis dataset (ERA5), provided in a gridded format with a spatial resolution of 0.1° × 0.1° and a temporal resolution of 1 hour (*65*).

2) The water quality factors encompass FAI (*25*) and hue angle (*a*) (*26*), which were computed using MODIS reflectance as follows

$$\begin{array}{c} \text{FAI}=R_{\text{rs}}\left(\text{NIR}\right)-R_{\text{rs}}\left(\text{red}\right)-\left[R_{\text{rs}}\left(\text{SWIR}\right)-R_{\text{rs}}\left(\text{red}\right)\right]\times \\ \left(859-645\right)/\left(1240-645\right) \end{array}$$ (1)

where *R*_rs_(red), *R*_rs_(NIR), and *R*_rs_(SWIR) represent the water leaving reflectance in the red, near-infrared, and short-wave infrared bands, respectively.

The hue angle (*a*′) was calculated from *R*_rs_(red), green reflectance [*R*_rs_(green)], and blue reflectance [*R*_rs_(blue)], as follows

$$a^{\prime }=a-\mathrm{\Delta a}$$ (2)

$$a=\left(\text{arctan}2\frac{x-0.33}{y-0.33}\right)\frac{180}{\pi }+180$$ (3)

$$x=\frac{X}{X+Y+Z}$$ (4)

$$y=\frac{Y}{X+Y+Z}$$ (5)

$$\begin{array}{c} \mathrm{\Delta a}=-1.8185{\left(a/100.0\right)}^{5}-62.461{\left(a/100.0\right)}^{4}+320.49{\left(a/100.0\right)}^{3}\\ -510.97{\left(a/100.0\right)}^{2}+306.32\left(a/100.0\right)-50.343 \end{array}$$ (6)

$$X=2.7689\times R_{\text{rs}}\left(\text{red}\right)+1.7517\times R_{\text{rs}}\left(\text{green}\right)+1.1302\times R_{\text{rs}}\left(\text{blue}\right)$$ (7)

$$Y=1.0000\times R_{\text{rs}}\left(\text{red}\right)+4.5906\times R_{\text{rs}}\left(\text{green}\right)+0.0601\times R_{\text{rs}}\left(\text{blue}\right)$$ (8)

$$Z=0.0565\times R_{\text{rs}}\left(\text{green}\right)+5.5943\times R_{\text{rs}}\left(\text{blue}\right)$$ (9)

3) Geographic factors encompass elevation and absolute latitude, which are static data that remain constant over time. Elevation were derived from a digital elevation model, with a resolution of 30 m by 30 m, sourced from COPERNICUS by the European Union and European Space Agency (ESA), accessible via the provided link (https://docs.sentinel-hub.com/api/latest/static/files/data/dem/resources/license/License-COPDEM-30.pdf). Latitude information is provided with the central latitude of each pixel and can be acquired using the “pixelLonLat()” method in GEE.

### Matchup generation

The matchups between three types of inputs (climate factors, water color factors, and geographic factors) and in situ DO were determined by the following criteria: (i) the time interval between ERA5 climate factors and in situ DO was restricted to within 1 hour; (ii) the time interval between water color factors and in situ DO was restricted to within 2 days; (iii) the geographic factors (e.g., elevation and latitude) are static data that remain constant over time. The mean value within a 3 × 3 pixels window centered at each sampling location was used as the matchup value. Only matchups within the window exhibiting a coefficient of variation of less than 10% were used (*62*, *66*). Last, a total of 32,810 valid matchups were obtained, of which 3.6% (1165) were recorded only once, while 96.4% were from time series data.

The matchup datasets were primarily conducted in North America, Europe, and Asia, with only a limited number of sample points distributed across Africa and other regions (table S1 and fig. S18). The matchup dataset is approximately evenly distributed across seasons (fig. S18C), showcasing a wide DO range from 0.4 to 20.0 mg/liter, with an average of 8.02 ± 2.39 mg/liter (fig. S18).

### Model development

We incorporated climate factors (air temperature, air pressure, solar radiation, thermal radiation, precipitation, and wind speed), water quality factors (FAI and hue angle), and geographic factors (elevation and absolute latitude value) that achieved better predictive performance into the model for global DO estimation in lakes and reservoirs. Temperature is considered the primary and ultimate cause of ongoing deoxygenation (*1*, *4*, *5*). Elevated temperatures reduce oxygen solubility (the capacity of water to hold oxygen) in water, increase the rate of oxygen consumption via respiration, and are predicted to diminish the vertical transportation of oxygen into deep water by enhancing stratification and weakening overturning circulation (*4*). The atmosphere pressure is another factor affecting oxygen solubility, with higher atmospheric pressure enabling more oxygen molecules to dissolve in the water (*3*, *67*). Underwater radiation, particularly solar radiation, notably influences oxygen supply through the photosynthesis of aquatic vegetation and phytoplankton (*13*). Precipitation alters DO concentration in regions strongly influenced by their watersheds (*68*) by carrying increasing loads of nutrients (nitrogen and phosphorus) and organic matter from the watershed. This process simultaneously promotes microbial respiration, increasing oxygen consumption (*15*), and stimulates algal growth, leading to higher algal biomass (*4*). Consequently, it contributes to both oxygen consumption and oxygen production (*5*, *14*). Wind maintains dynamic equilibrium in DO levels through air-water reoxygenation (*69*). Elevation was incorporated into the model as it provides insights into climate patterns, vegetation distribution, and hydrological processes, all of which intricately influence the long-term average DO values at sites with varying elevations. On the other hand, absolute latitude denotes the geographic location of the study area relative to the equator. It reflects solar radiation intensity and seasonal fluctuations, subsequently influencing water temperature and photosynthetic activity within aquatic ecosystems. By incorporating elevation and latitude as model input parameters, the model can effectively encapsulate these topographic nuances, thereby enhancing its capacity to simulate DO dynamics across varying altitudes and geographical positions. The FAI provides insights into phytoplankton biomass, where elevated FAI values indicate dense aggregations of surface phytoplankton (*27*); while hue angle stands as a straightforward metric for assessing trophic status (*28*). Both FAI and hue angle serve as valuable indicators of eutrophication.

In SEM, the importance of each variable is assessed through standardized path coefficients (*70*). These coefficients indicate the strength and direction of the relationships between variables, allowing for comparisons of their relative contributions. The standardized coefficients are regression coefficients calculated by using standardized variables as inputs in the regression model. Standardization ensures that all variables have a mean of 0 and an SD of 1, enabling direct comparison of their effects. As a result, the coefficients represent the change in the dependent variable for a one-SD change in the independent variable, regardless of the original units of measurement. The SEM was implemented in R and fitted using the “piecewiseSEM” package (https://cran.r-project.org/web/packages/piecewiseSEM/vignettes/piecewiseSEM.html). The importance analysis showed that the foremost determinant factor is the climate factor, particularly air temperature and solar radiation, contributing to −50 and 19% of the variation in DO concentration, respectively. This is followed by geographic factors, especially elevation, which contributes to 27%, and then water quality represented by the FAI, which contributes −13%. The specifics regarding the variable importance for the inputs are depicted in fig. S1A.

The state-of-the-art algorithms in the previous studies suggest that machine learning methods can capture nonlinear relationships between input features and biophysical parameters or inherent optical properties and generally show improved performance over simple regression methods (*71**–**75*). We selected five commonly used machine learning methods, i.e., back-propagation (BP) neural network, support vector regression (SVR), RFs, extreme gradient boosting (XGBoost), and long short-term memory (LSTM), as the candidate models to estimate DO concentration (fig. S1B and Supplementary Text 2). BP and SVR are single models, each comprising only one basic learning algorithm, making them simpler and easier to be interpreted. On the other hand, RF and XGBoost are ensemble learning models that amalgamate multiple basic learning algorithms to construct a robust model, which provide better performance when dealing with more complex datasets and generally improve prediction accuracy. LSTM networks are a type of recurrent neural network that is designed to address the issue of learning long-term dependencies in sequential data (*76*). Their unique architecture enables them to effectively retain information over extended sequences. The details of these models are described in Supplementary Text 2. A total of 22,967 randomly chosen matchups were used for training the model, leaving out the remainder of the data (*N* = 9843) for testing. All input data were standardized using the Standard Scaler in the scikit-learn package and then scaled to the 0 to 1 range. By defining a hyperparameter grid and conducting a grid search method spanning more than 100 epochs for each model, an optimal parameter list was derived, as detailed in table S2.

Among these models, the RF model demonstrated satisfactory accuracy on the training dataset, yielding an average mean absolute error (MAE) of 0.72 mg/liter, mean relative error (MRE) of 10%, root mean square error (RMSE) of 0.95 mg/liter, and a coefficient of determination (*R*^2^) of 0.84. Furthermore, the testing results for the RF model also demonstrated high performance, with an average MAE of 0.95 mg/liter, MRE of 13%, RMSE of 1.35 mg/liter, and *R*^2^ of 0.72 (fig. S2). The XGBoost model exhibited the second-best performance, achieving an average MAE of 0.74 mg/liter, MRE of 10%, RMSE of 1.03 mg/liter, and *R*^2^ of 0.82 on the training dataset and an average MAE of 0.98 mg/liter, MRE of 13%, RMSE of 1.39 mg/liter, and *R*^2^ of 0.66 on the testing dataset. Analysis of the unity line for the RF model indicated a well-distributed dispersion of data pairs. Furthermore, the RF model outperformed SVR, BP, LSTM, and XGBoost in providing accurate estimates for both low and high DO values. Consequently, the RF model was selected as the final model for DO estimation.

### Time series analysis

For long-term DO analysis, we focus on the summer months (July to September in the Northern Hemisphere and January to March in the Southern Hemisphere) in each year to get snow/ice-free data. This temporal focus has been previously validated, demonstrating reduced susceptibility to snow/ice interference (*77*, *78*). To mitigate the impact of outliers, we used the median in region and for each year as robust estimators of the mean (*79*). Long-term trends spanning from 2003 to 2023 in DO were computed using the Theil-Sen median method implemented in the SciPy package (*80*). Significance of slopes was tested through a Mann-Kendall test (*81*, *82*), designed to identify monotonic trends and widely used in detecting water color trends (*83*).

We performed a comprehensive analysis of the seasonal variation in DO across global lakes, spanning from January to December. A dynamic NDWI approach, as detailed in the “Lake water identification” section, was used to enhance the accuracy of our analysis. In addition, a temperature mask was applied to exclude pixels with air temperatures below 0°C, thereby mitigating the potential influence of snow or ice on the DO estimation. Seasonal DO fluctuation was determined by subtracting the minimum monthly mean DO from the maximum monthly mean DO. In addition, we conducted comparative analyses of DO seasonal variations between the Southern and Northern Hemispheres to elucidate regional disparities.

### Impact of long-term climatic change to DO variation

The SEM was used to quantify the contributions of long-term climate changes—specifically temperature, wind speed, and atmospheric pressure—to variations in solubility. In addition, SEM assessed the impacts of long-term solubility changes and eutrophication on DO variations across global lakes. The time series of solubility, DO, FAI, and various climatic drivers (21 annual means from 2003 to 2023) were normalized before being used in the SEM model. Since temperature, wind speed, and atmospheric pressure can indirectly affect DO concentration by altering solubility, this indirect influence is represented by arrows from temperature, wind speed, and air pressure pointing to solubility and then to DO concentration. In addition, temperature can also indirectly influence DO concentration by modifying eutrophication, represented by arrows from temperature to FAI and subsequently to DO concentration.

### Heatwave definition and its impact on DO

In the absence of daily lake surface water temperature data, we used ERA5 daily air temperature data (*65*) to identify atmospheric heatwave events over global lakes during 2003–2023 period and evaluate their impacts on lake surface deoxygenation. Atmospheric heatwaves were identified when daily atmospheric temperatures exceeded a threshold and persisted for several days (*72*–*74*, *84*, *85*). The threshold was defined as the 90th percentile value for each calendar day within the climatological period (1970–1999) (*84*). A percentile-based threshold facilitates comparisons of heatwaves across locations with differing variability. Using a “day-specific” threshold enables the detection and measurement of events throughout the year, accommodating variations such as winter heatwaves with lower temperature than those occurring in summer (*84*, *85*). According to this definition, the 90th percentile threshold had to be exceeded for at least three consecutive days to be considered a heatwave event (*84*). However, for global-scale analysis, we follow the recommendations of a 5-day exceedance condition (*22*, *85*). Heat spikes lasting fewer than 5 days were not considered heatwave events (*85*).

To evaluate the impact of heatwaves on DO concentrations, we quantified the influence intensity as the mean percentage relative difference between two DO values generated by our constructed RF model. The model inputs include three categories of data: climate factors (air temperature, air pressure, solar radiation, thermal radiation, precipitation, and wind speed), water quality factors (FAI and hue angle), and geographic factors (elevation and absolute latitude). The distinction lies in the temperature data, which were actual temperature during heatwave periods and climatological mean temperature within the period of 1970–1999, respectively. The mean influence of intensity reflects the average intensity during heatwave events, while the maximum influence intensity indicates the peak intensity observed during an event.

To assess the impact of heatwaves on long-term lake deoxygenation, we used a constructed RF model to calculate the long-term variation in DO concentrations under two scenarios: one with actual temperatures, including extreme heatwave events, and another excluding heatwave events. We then compared the mean values and the rate of change between these two DO metrics. The relative difference between these two DO metrics, normalized by the value under actual temperature conditions, defines the impact of heatwaves on long-term lake deoxygenation.

### Definition of DO stress

Stress induced by declining DO levels in lake ecosystems may lead to sublethal negative health effects for freshwater fish populations. Different fish species have different DO tolerance. For example, the growth of *Huso huso* and *Ictalurus punctatus*, both categorized as shallow-water fish, is adversely affected when DO concentrations decrease to 3.95 and 6.37 mg/liter, respectively. Similarly, *Oncorhynchus mykiss*, another shallow-water species, experiences impaired growth and food consumption at DO levels below 5.46 mg/liter. Furthermore, reduced growth and food intake are observed in *Morone saxatilis* x *Morone chrysops* when DO levels drop to 7.0 and 5.69 mg/liter. On average, the thresholds for growth reduction and decreased food consumption are found to be 5.1 and 5.6 mg/liter, respectively (*30*). This study quantifies DO stress conditions, defined as DO levels below 6.0 mg/liter, at which fish growth and food consumption notably decline in freshwater ecosystems (*30*). The stress frequency refers to the percentage frequency of occurrences when the average DO is below 6 mg/liter. The stress lake was the lake with the annual average DO below 6.0 mg/liter.

### Future DO projections

Three types of variables served as inputs for the model to project future DO trends. The first type comprises static inputs, including elevation and absolute latitude, which remain constant over time. The second type encompasses projected daily climate variables, such as solar radiation, thermal radiation, air temperature, air pressure, precipitation, and wind speed. These climate variables were sourced from eight CMIP6 (Coupled Model Intercomparison Project Phase 6) models representing two SSP scenarios: SSP2-4.5 and SSP5-8.5. The third type of variable, not available from optical satellites (such as, FAI and hue angle), was replaced by mean values observed over the most recent 5 years (2019–2023).

## Supplementary Materials

### This PDF file includes:

Supplementary Texts 1 and 2 Figs. S1 to S20 Tables S1 and S2 References
