# Supplementary material for: Climate warming and heatwaves accelerate global lake deoxygenation
Source: Sci Adv. 2025 Mar 21;11(12):eadt5369. doi: 10.1126/sciadv.adt5369 (PMC11927634; doi:10.1126/sciadv.adt5369)
Supplement: Supplementary file 1 — Supplementary Texts 1 and 2 Figs. S1 to S20 Tables S1 and S2 References [file sciadv.adt5369_sm.pdf]

Science Advances
AAAS

Supplementary Materials for

Climate warming and heatwaves accelerate global lake deoxygenation

Yibo Zhang *et al.*

Corresponding author: Kun Shi, kshi@niglas.ac.cn; Yunlin Zhang, ylzhang@niglas.ac.cn

Sci. Adv. **11**, eadt5369 (2025)
DOI: 10.1126/sciadv.adt5369

This PDF file includes:

Supplementary Texts 1 and 2
Figs. S1 to S20
Tables S1 and S2
References

Supplementary Text

1. DO saturation percentage increased over time despite reduced gas solubility

The determination of DO solubility (DOsol) relies on a reference lookup using temperature and air pressure (Fig. S19). The annual mean DOsol of global lakes from 2003 to 2023 is estimated to be 10.24±1.42 mg/L (Fig. S5a and S5b). The spatial distribution of DOsol contrasts with that of air temperature (Fig. S20). Specifically, North America and Europe exhibit substantially higher DOsol than other continents, boasting multiyear averages of 10.62±1.20 mg/L and 10.65±1.08 mg/L, respectively (Fig. S5a and S5e). The spatial distribution and regional dynamics of the DOsol rate are detailed in Fig. S5c and S5f. Globally, lakes exhibit an overall decreasing trend in DOsol, with a mean rate of -0.12 mg/L/decade during the study period (Fig. S5d). Moreover, DOsol has decreased across all six continents, with declines of -0.039 mg/L in Africa, -0.15 mg/L in Asia, -0.21 mg/L in Europe, -0.32 mg/L in North America, -0.12 mg/L in Oceania, and -0.068 mg/L in South America, respectively over the past two decades (Fig. S5c and S5f).

The DO saturation percentage (DO SP), representing the percentage of DO concentration versus DOsol (Fig. S6), captures biogeochemical controls, such as photosynthesis and respiration, beyond temperature effects. The annual mean DO SP of global lakes from 2003 to 2023 is estimated to be 93.84±7.63 % (Fig. S6a and S6b). Notably, Asia, Europe, North America and Oceania exhibit multiyear averages of 93.03±13.08 %, 94.19±5.05 %, 94.88±5.29 % and 93.99±9.07 % respectively, showing significantly higher DO SP than Africa (85.66±11.77 %) and South America (87.93±11.10 %) (Fig. S6a and S6e). The spatial distribution and regional dynamics of the DO SP rate are outlined in Fig. S6c and S6f. Specifically, the DO SP has

increased across all six continents over the past two decades, with increments of 0.07% in Africa, 1.25% in Asia, 0.75% in Europe, 0.87% in North America, 0.67% in Oceania, and 0.61% in South America, respectively (Fig. S6c and S6f). Globally, 68% of the studied lakes exhibit an increase in DO SP, with an average rate of 0.86%/decade, potentially indicating enhanced photosynthesis (oxygen supply) and/or reduced aquatic respiration (oxygen consumption) in these areas. Meanwhile, 32% of the studied lakes experienced a decrease in DO SP, averaging -0.52%/decade (Fig. S6g), potentially indicating declining photosynthesis or accelerated aquatic respiration. Overall, lake DO SP worldwide increased by 0.88% in the past two decades (t-test, slope = 0.044 %/decade, $P < 0.01$) (Fig. S6d and S6h).

2. Candidate models to estimate DO concentrations

We selected five commonly used machine learning methods, i.e., back propagation neural network (BP), support vector regression (SVR), random forests (RF), extreme gradient boosting (XGBoost) and long short-term memory (LSTM), as the candidate models to estimate DO concentration in lakes.

BP is a supervised learning algorithm used for training artificial neural networks [86]. It is based on minimizing the error between the predicted output and the actual target by adjusting the weights (W^{IH}) of the network through backpropagation. The process works by passing input data (X^1, X^1,…,X^N) through the network, applying an activation function (e.g., relu, tanh, Sigmoid) (Fig. S1) at each hidden layer to introduce non-linearity, and calculating the output (DO_i). After calculating the output, the prediction is compared to the true value, and the weights are updated using gradient descent to reduce the prediction error. This process continues iteratively until the model meets the stopping criterion. BP is particularly effective for learning

complex non-linear relationships between inputs and outputs, making it a useful tool for modeling DO concentration.

SVR is a type of regression technique that extends the concept of Support Vector Machines (SVM) to regression problems [87]. In SVR, the goal is to find a function that approximates the target values as closely as possible, within a specified margin of error. The input features X^1, X^1,…,X^N are transformed into a higher-dimensional feature space using a kernel function, which allows the model to learn non-linear relationships between the input data and the DO concentration. SVR aims to find a balance between fitting the training data well and keeping the model as simple as possible (minimizing the complexity of the regression function). The kernel function (e.g., linear, rbf, sigmoid, poly function) (Fig. S1) is chosen based on the nature of the data, and the complexity of the model is controlled by a regularization parameter. SVR is particularly useful in cases where the data contains noise or where the relationship between the features and the target is complex and non-linear.

RF is an ensemble learning technique that builds multiple decision trees and merges them together to improve the accuracy and robustness of predictions [88]. Each tree is trained on a random subset of the training data, and predictions are made by aggregating the results (DO_i) from all trees, typically by averaging for regression tasks (Fig. S1). RF is particularly effective at handling large datasets with numerous features and is resilient to overfitting. Additionally, it provides feature importance, which is valuable for identifying the most influential variables in predicting lake DO.

XGBoost is a highly efficient and scalable implementation of gradient boosting [89], a technique that builds a series of decision trees, where each new tree corrects the errors made by

the previous one. It uses an ensemble of weak learners (decision trees) to form a strong predictive model. XGBoost improves on traditional gradient boosting by incorporating regularization techniques that reduce overfitting and improve performance. XGBoost has become popular due to its high accuracy, speed, and scalability, making it a powerful tool for large datasets.

LSTM, a deep learning model, leverages memory cells and information gates—including input, forget, and output gates—to efficiently store and transmit information across different time steps through vector operations (i.e., scale and add) [76]. In particular, the model's inputs comprise the current input (X_t), memory from previous LSTM unit (C_{t-1}) and output from previous LSTM unit (h_{t-1}). The model's outputs comprise the new updated memory (C_t) and current output (h_t). The input gate, represented by a sigmoid function, determines which information needs to be updated, while the forget gate determines which information should be retained or discarded. Finally, the output gate determines the next hidden state, encapsulating information from previous inputs and facilitating predictions (Fig. S1). Our LSTM architecture consists of a single input layer, a hidden layer containing four LSTM blocks, and an output layer responsible for generating a single prediction. The activation function is employed with sigmoid method, and optimization of the loss function is achieved through the Adaptive Moment Estimation (ADAM) optimization algorithm.

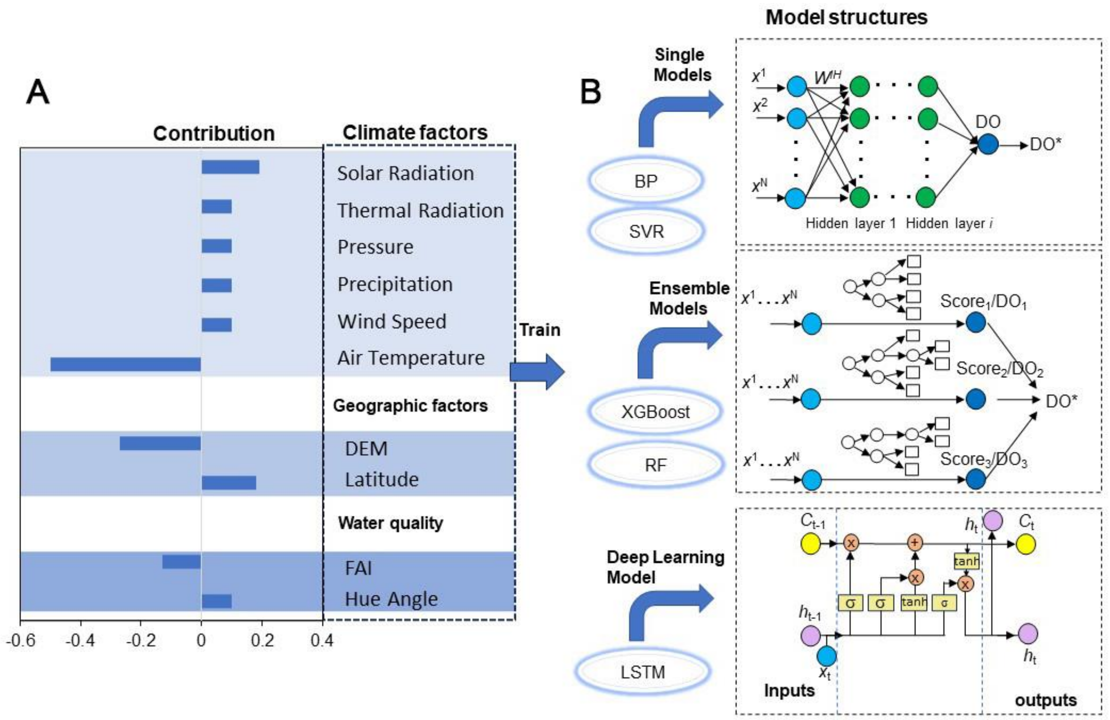

Fig. S1. The structure and inputs of the five data-driven models. (A) Model training includes importance analysis using structural equation modeling (SEM) to assess the contribution of each variable to changes in DO concentration [70]. (**B**) Model structure design. The foremost determinant factor is the climate factor, particularly air temperature and solar radiation, contributing to -50% and 19% of the variation in DO concentration, respectively. This is followed by geographic factors, especially the elevation, which contributes to 27%, and then water quality represented by the FAI, which contributes to -13%. Two single models (BP and SVR), two ensemble models (XGBoost and RF) and one deep learning model (LSTM) were used as the candidate models to estimate DO, and the one with best accuracy in fig. S2 was selected as the final model to estimate DO in this research.

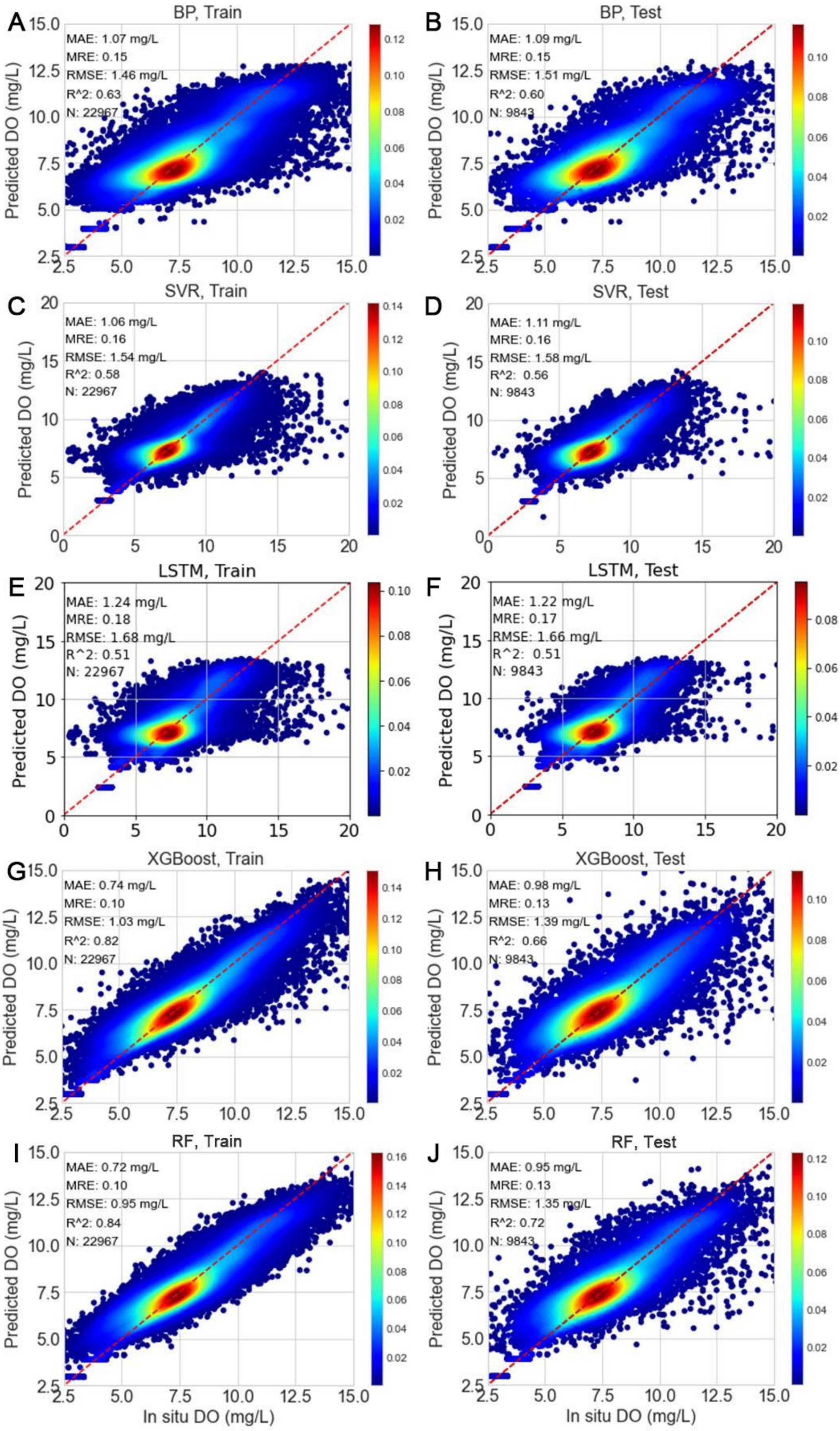

A
BP, Train
MAE: 1.07 mg/L
MRE: 0.15
RMSE: 1.46 mg/L
R^2: 0.63
N: 22967
B
BP, Test
MAE: 1.09 mg/L
MRE: 0.15
RMSE: 1.51 mg/L
R^2: 0.60
N: 9843
C
SVR, Train
MAE: 1.06 mg/L
MRE: 0.16
RMSE: 1.54 mg/L
R^2: 0.58
N: 22967
D
SVR, Test
MAE: 1.11 mg/L
MRE: 0.16
RMSE: 1.58 mg/L
R^2: 0.56
N: 9843
E
LSTM, Train
MAE: 1.24 mg/L
MRE: 0.18
RMSE: 1.68 mg/L
R^2: 0.51
N: 22967
F
LSTM, Test
MAE: 1.22 mg/L
MRE: 0.17
RMSE: 1.66 mg/L
R^2: 0.51
N: 9843
G
XGBoost, Train
MAE: 0.74 mg/L
MRE: 0.10
RMSE: 1.03 mg/L
R^2: 0.82
N: 22967
H
XGBoost, Test
MAE: 0.98 mg/L
MRE: 0.13
RMSE: 1.39 mg/L
R^2: 0.66
N: 9843
I
RF, Train
MAE: 0.72 mg/L
MRE: 0.10
RMSE: 0.95 mg/L
R^2: 0.84
N: 22967
J
RF, Test
MAE: 0.95 mg/L
MRE: 0.13
RMSE: 1.35 mg/L
R^2: 0.72
N: 9843
Predicted DO (mg/L)
In situ DO (mg/L)

Fig. S2. Scatterplots of the measured vs. estimated DO based on the BP, SVR, LSTM, XGBoost and RF algorithms. (**A-B)** Model training and testing based on BP algorithm. (**C-D)** Model training and testing based on SVR algorithm. (**E-F)** Model training and testing based on LSTM algorithm. (**G-H)** Model training and testing based on XGBoost algorithm. **(I-J)** Model training and testing based on RF algorithm. The density plots are calculated using a Gaussian kernel density estimator.

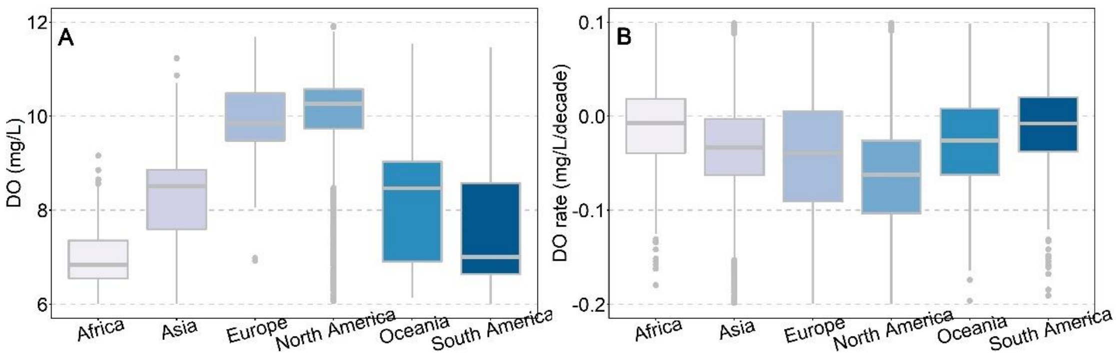

Fig. S3. Boxplots of DO concentration for different continents. (A) Boxplots representing the mean DO concentration for the continents of Africa, Asia, Europe, North America, Oceania, and South America. (**B**) Boxplots representing the change rate of DO concentration for the continents. Within these boxplots, the median values are depicted by the middle lines, the 75th and 25th percentiles are denoted by the maximum and minimum extents of the colored boxes, while the whiskers represent the 5th and 95th percentiles, respectively.

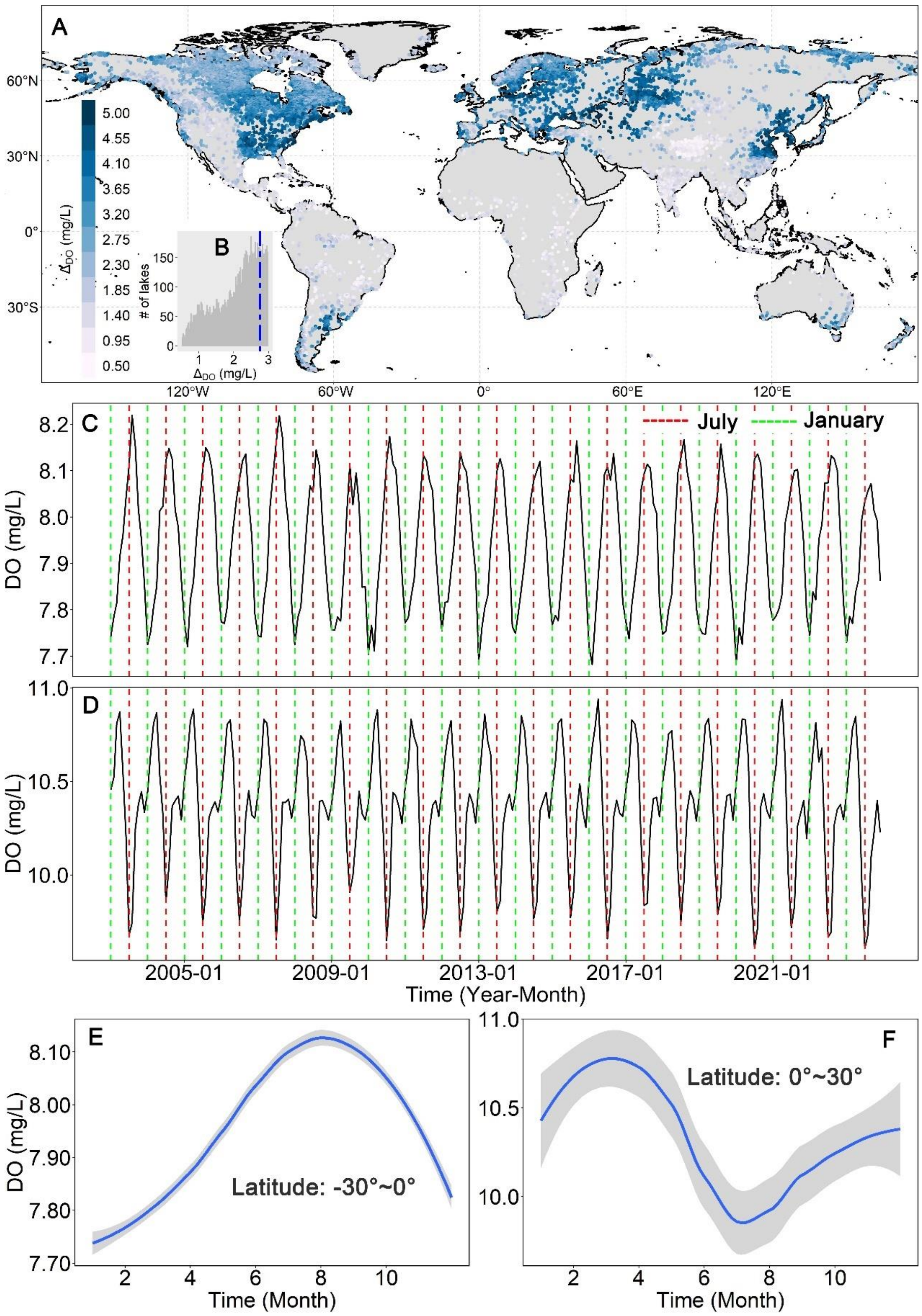

Fig. S4. Seasonal variations of DO concentrations in global lakes. (**A**) DO concentrations exhibit large fluctuations throughout the year. (**B)** Statistical histograms illustrating DO fluctuations throughout the year, with a vertical blue line representing the mean value. (**C**)

Long-term variation of global DO shows a peak around August and a trough around January in the Southern Hemisphere. (**D**) A different trend is observed in the Northern Hemisphere, with a peak around March and a trough around July. (**E**) The monthly averaged DO in the Southern and Northern Hemisphere exhibits contrasting trends. The Southern Hemisphere exhibits a convex shape. (**F**) The Northern Hemisphere displays a combination of both convex and concave patterns.

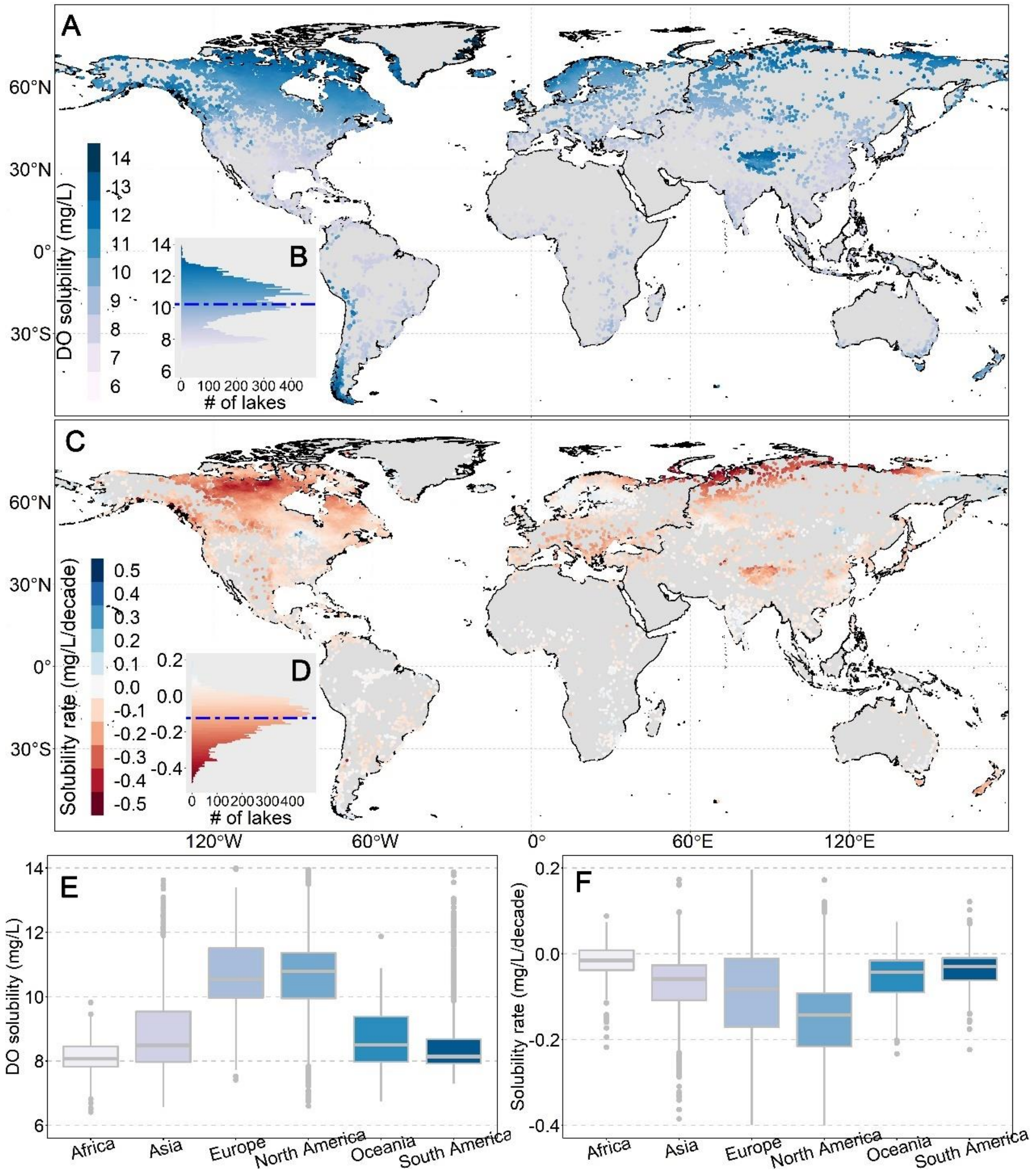

Fig. S5. Spatiotemporal variations in DO solubility (DOsol) for global lakes with surface areas ≥ 10 km^2. (**A**) The spatial distribution of the mean DOsol is delineated for global lakes. (**B**) Statistical histograms illustrating the mean DOsol, with horizontal dashed lines representing the mean values. (**C**) The spatial distribution of the change rate of DOsol for global lakes. (**D**) Statistical histograms illustrating the change rate of DOsol, with horizontal dashed lines representing the mean values. (**E**) Boxplots representing the mean DOsol across the continents of Africa, Asia, Europe, North America, Oceania, and South America.

(F) Boxplots representing the change rate of DOsol across the continents of Africa, Asia, Europe, North America, Oceania, and South America. Within these boxplots, the median values are depicted by the middle lines, the 75th and 25th percentiles are denoted by the maximum and minimum extents of the colored boxes, while the whiskers represent the 5th and 95th percentiles, respectively.

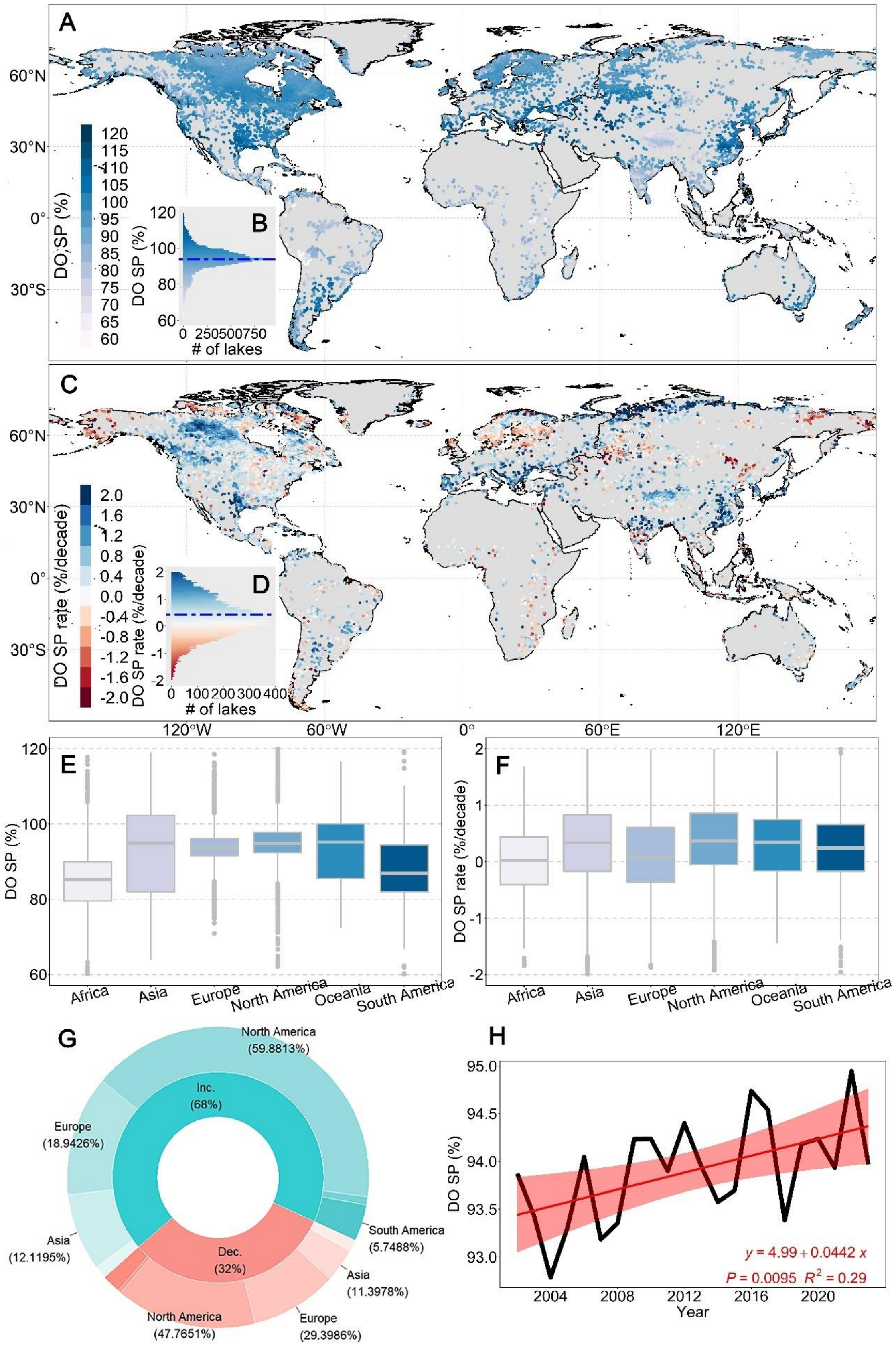

Fig. S6. Spatiotemporal variations in DO saturation percentage (DO SP) for global lakes with surface areas ≥ 10 km^2. (**A**) The spatial distribution of the mean DO SP for global lakes

with areas ≥ 10 km². (**B**) Statistical histograms illustrating the mean DO SP, with horizontal dashed lines representing the mean values. (**C**) The spatial distribution of the change rate of DO SP for global lakes. (**D**) Statistical histograms illustrating the change rate of DO SP, with horizontal dashed lines representing the mean values. (**E**) Boxplots representing the mean DO SP across the continents of Africa, Asia, Europe, North America, Oceania, and South America. (**F**) Boxplots representing the change rate of DO SP across the continents of Africa, Asia, Europe, North America, Oceania, and South America. (**G**) Statistical information of DO SP rate across two categories, namely increase and decrease, for each continent (Africa, Asia, Europe, North America, Oceania, and South America). (**H**) Decadal trends spanning from 1986 to 2021 for the mean DO on a global scale. Within these boxplots, the median values are depicted by the middle lines, the 75th and 25th percentiles are denoted by the maximum and minimum extents of the colored boxes, while the whiskers represent the 5th and 95th percentiles, respectively.

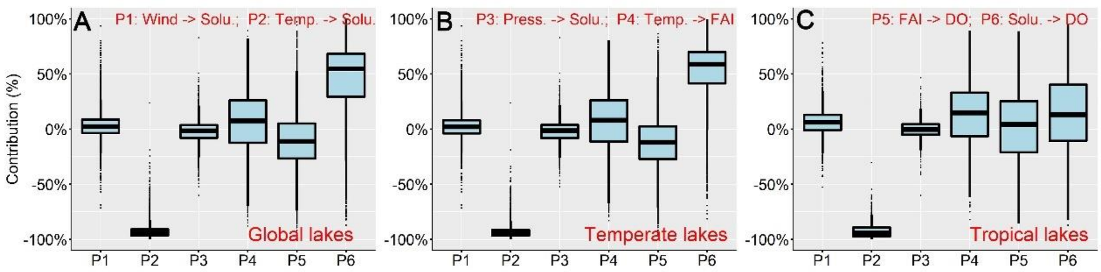

Fig. S7. Box plots illustrate the contributions of different pathways based on the SEM model for global lakes. (**A**) Global lakes. (**B**) Temperate lakes. (**C**) Tropical lakes. The pathways P1, P2, P3, P4, P5 and P6 correspond to the following relationships: P1: Wind -> Solubility, P2: Temperature -> Solubility, P3: Pressure -> Solubility, P4: Temperature -> FAI, P5: FAI -> DO, and P6: Solubility -> DO. Within these boxplots, the median values are depicted by the middle lines, the 75th and 25th percentiles are denoted by the maximum and minimum extents of the colored boxes, while the whiskers represent the 5th and 95th percentiles, respectively.

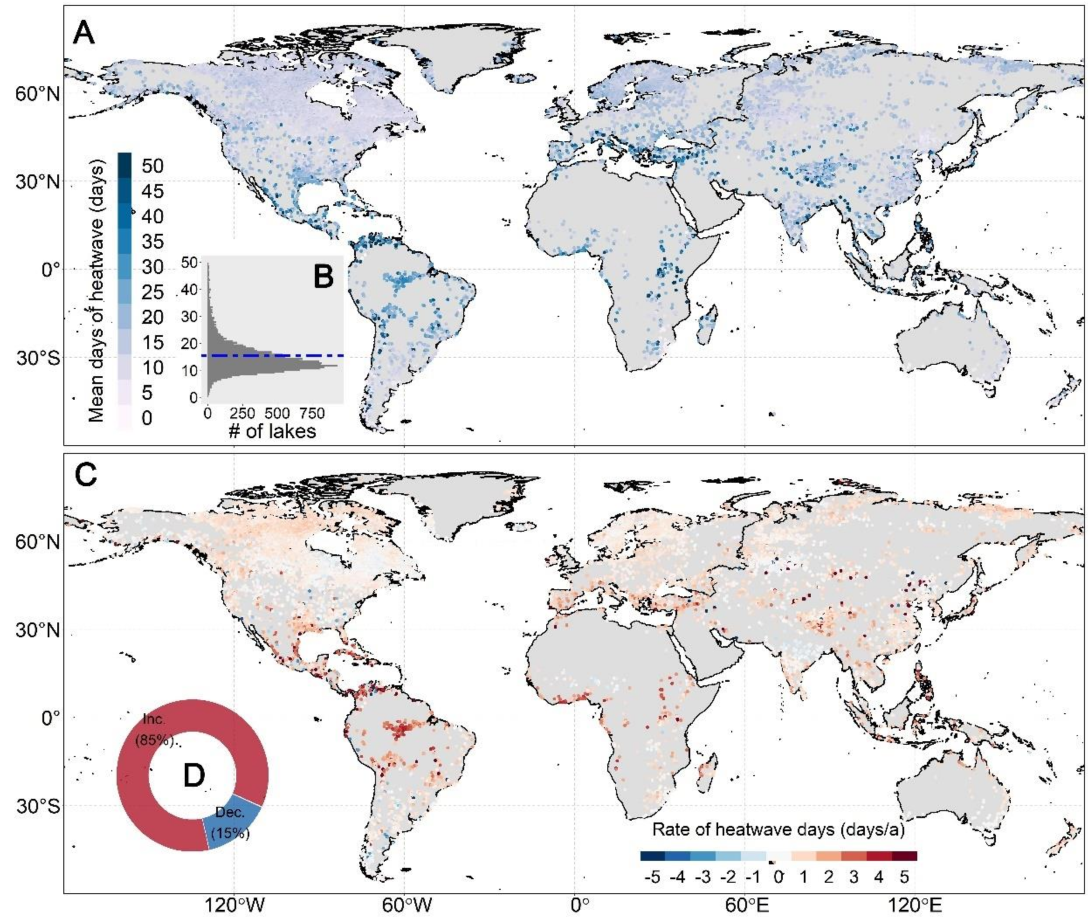

Fig. S8. Spatial-temporal variation of heatwave duration for global lakes with areas ≥10 km2. (**A**) The spatial distribution of the mean value of heatwave duration. (**B**) Statistical histograms illustrating the mean heatwave duration, with horizontal dashed lines representing mean values. (**C**) The spatial distribution of the change rate of heatwave duration. (**D**) Statistics on the number of lakes with increasing and decreasing heatwaves for global lakes with areas ≥10 km².

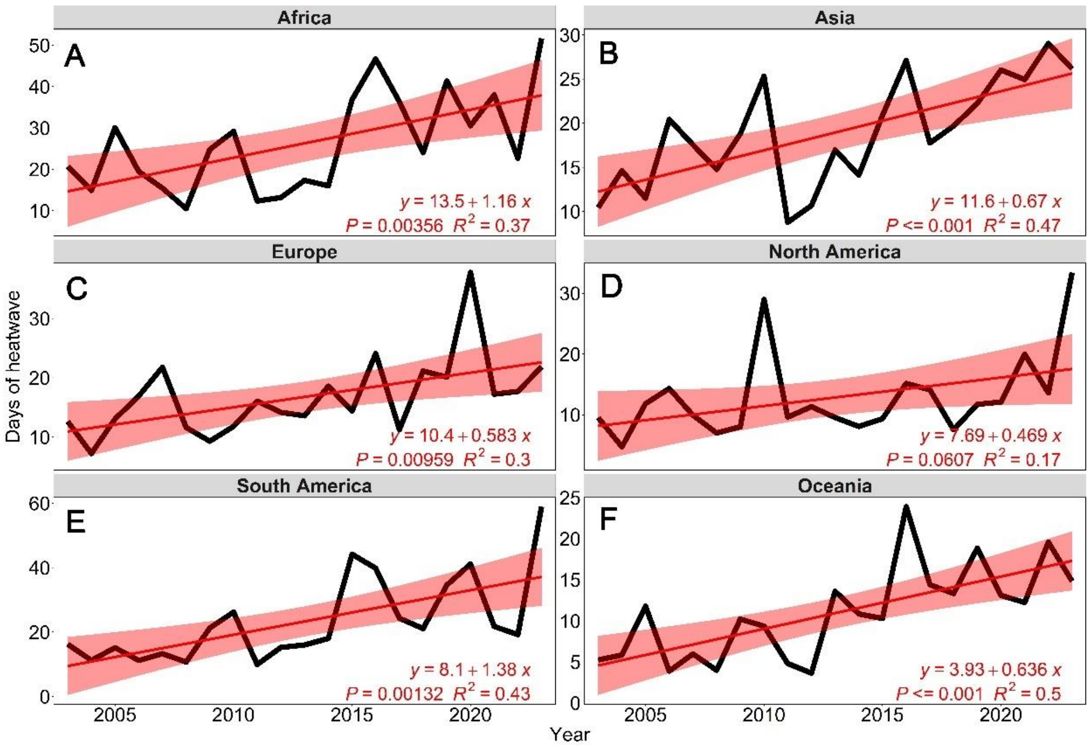

Fig. S9. **Long-term trends in heatwave duration across continents.** (**A**) Trends in heatwave duration for Africa. (**B**) Trends in heatwave duration for Asia. (**C**) Trends in heatwave duration for Europe. (**D**) Trends in heatwave duration for North America. (**E**) Trends in heatwave duration for South America. (**F**) Trends in heatwave duration for Oceania.

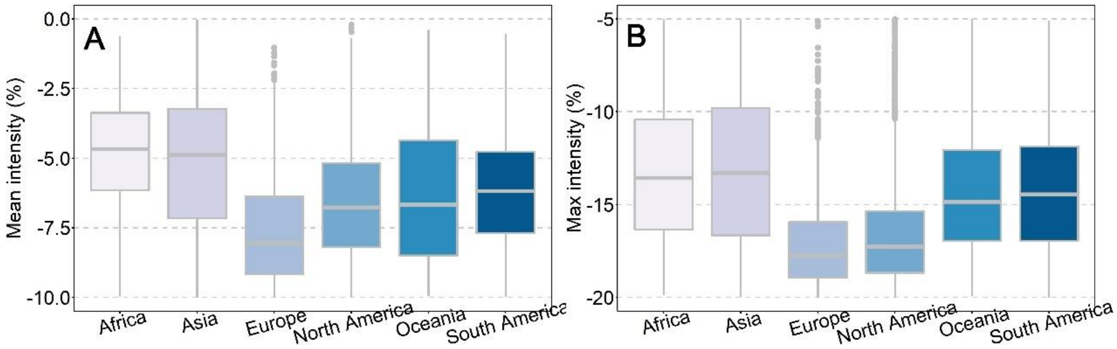

Fig. S10. Boxplots of heatwave influence intensity on lake deoxygenation across continents. (**A**) Boxplots representing the mean influence intensity for Africa, Asia, Europe, North America, Oceania, and South America. (**B**) Boxplots representing the maximum influence intensity for Africa, Asia, Europe, North America, Oceania, and South America. Within these boxplots, the median values are depicted by the middle lines, the 75th and 25th percentiles are denoted by the maximum and minimum extents of the colored boxes, while the whiskers represent the 5th and 95th percentiles, respectively.

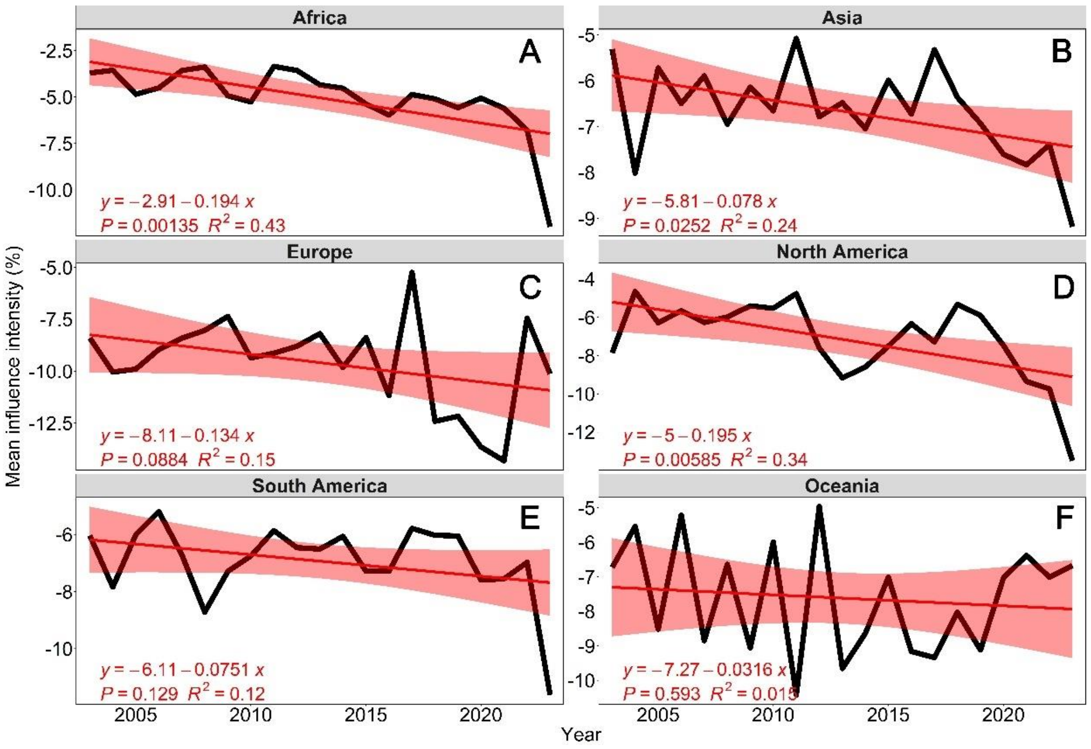

Fig. S11. Long-term trends in the mean influence intensity of heatwaves on DO concentration across continents. (**A**) Trend in mean influence intensity for Africa. (**B**) Trend in mean influence intensity for Asia. (**C**) Trend in mean influence intensity for Europe. (**D**) Trend in mean influence intensity for North America. (**E**) Trend in mean influence intensity for South America. (**F**) Trend in mean influence intensity for Oceania.

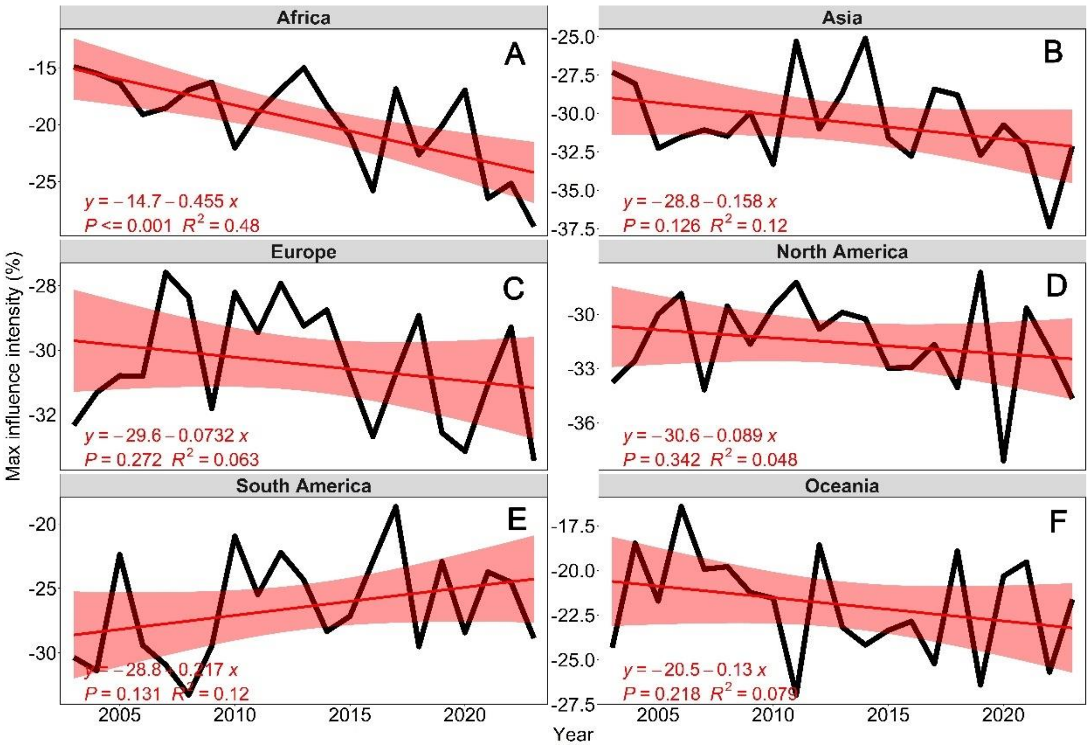

Fig. S12. Long-term trends in the maximum influence intensity of heatwaves on DO concentration across continents. (**A**) Trend in maximum influence intensity for Africa. (**B**) Trend in maximum influence intensity for Asia. (**C**) Trend in maximum influence intensity for Europe. (**D**) Trend in maximum influence intensity for North America. (**E**) Trend in maximum influence intensity for South America. (**F**) Trend in maximum influence intensity for Oceania.

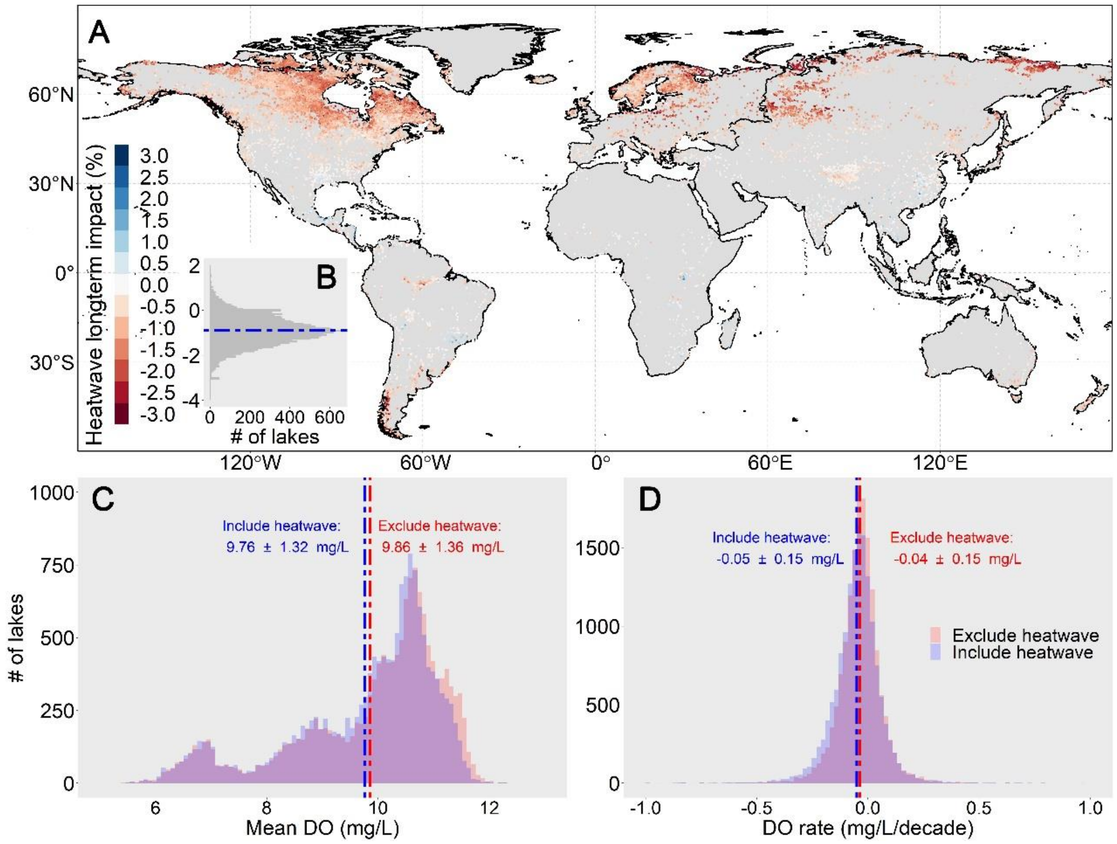

Fig. S13. Impact of heatwaves on long-term lake deoxygenation. (A) Spatial distribution of heatwave impacts on long-term lake deoxygenation, calculated using RF-estimated values under two scenarios: (1) driven by actual temperatures, including extreme heatwave events, and (2) driven by temperatures excluding heatwave events. The relative difference between these two DO metrics, normalized by the value under actual temperature conditions (including extreme heatwave events), defines the impact of heatwaves on long-term lake deoxygenation. (**B**) Statistical histograms showing the impacts of heatwaves on long-term lake deoxygenation, with horizontal dashed lines indicating median values. (**C**) Histogram distributions of mean DO under the two scenarios. (**D**) Histogram distributions of DO rate of change under the two scenarios.

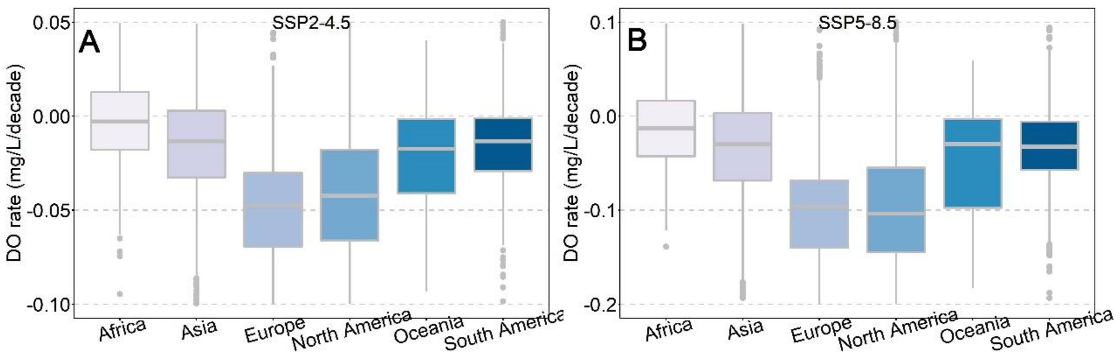

Fig. S14. **Boxplots of projected DO rates across continents.** (**A**) Boxplots representing the projected DO rates under SSP2-4.5 for the continents of Africa, Asia, Europe, North America, Oceania, and South America. (**B**) Boxplots representing the projected DO rates under SSP5-8.5 for the same continents. Within these boxplots, the median values are depicted by the middle lines, the 75th and 25th percentiles are denoted by the maximum and minimum extents of the colored boxes, while the whiskers represent the 5th and 95th percentiles, respectively.

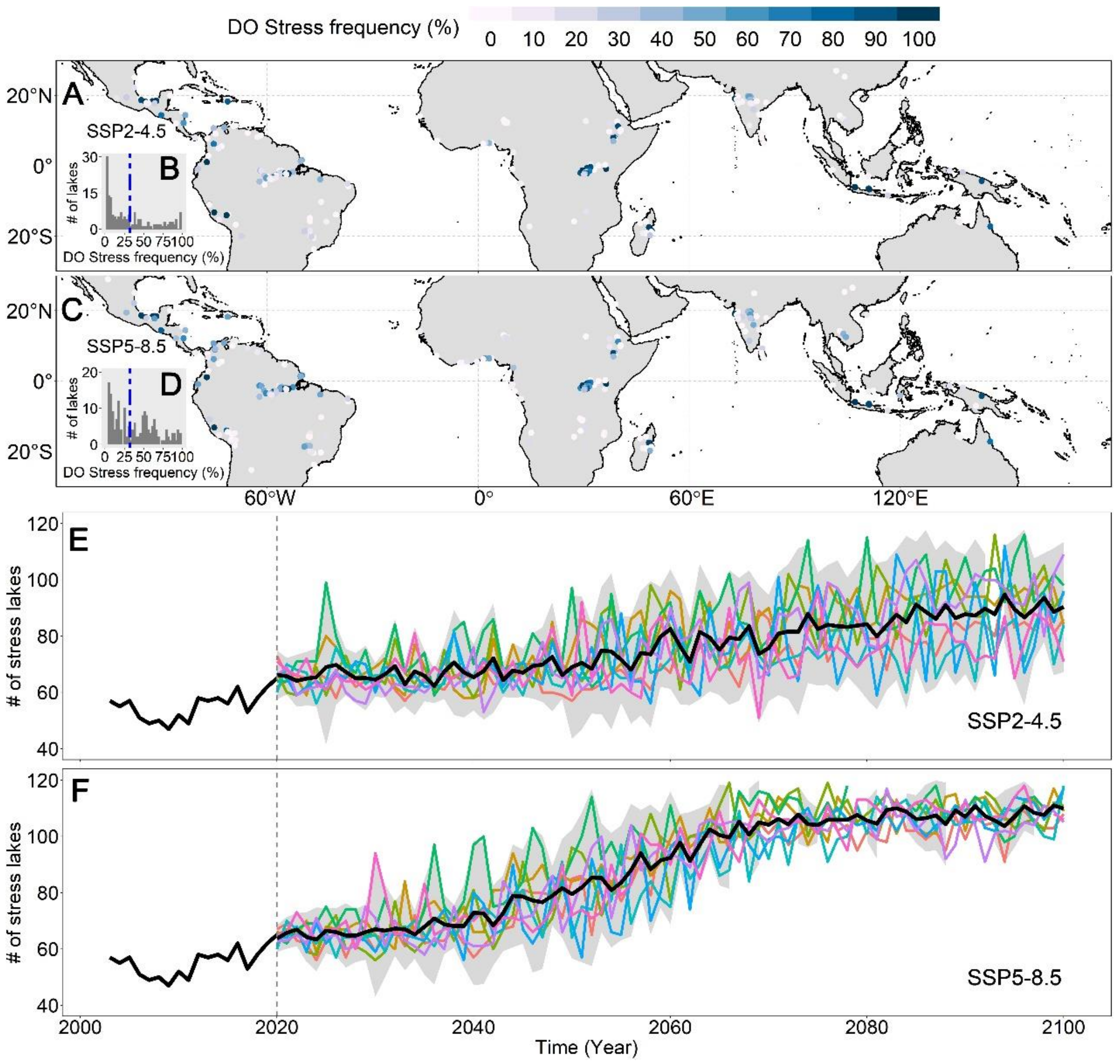

Fig. S15. **Projected DO stress under SSP2–4.5 and SSP5–8.5 scenarios.** (**A**) Spatial distribution of DO stress frequency under SSP2–4.5. (**B**) Spatial distribution of DO stress frequency under SSP5–8.5. (**C**, **D**) Statistical histograms illustrating DO stress frequency in the two scenarios, with vertical dashed lines representing mean values. (**E**, **F**) Projected number of stressed lakes under SSP2–4.5 and SSP5–8.5 scenarios.

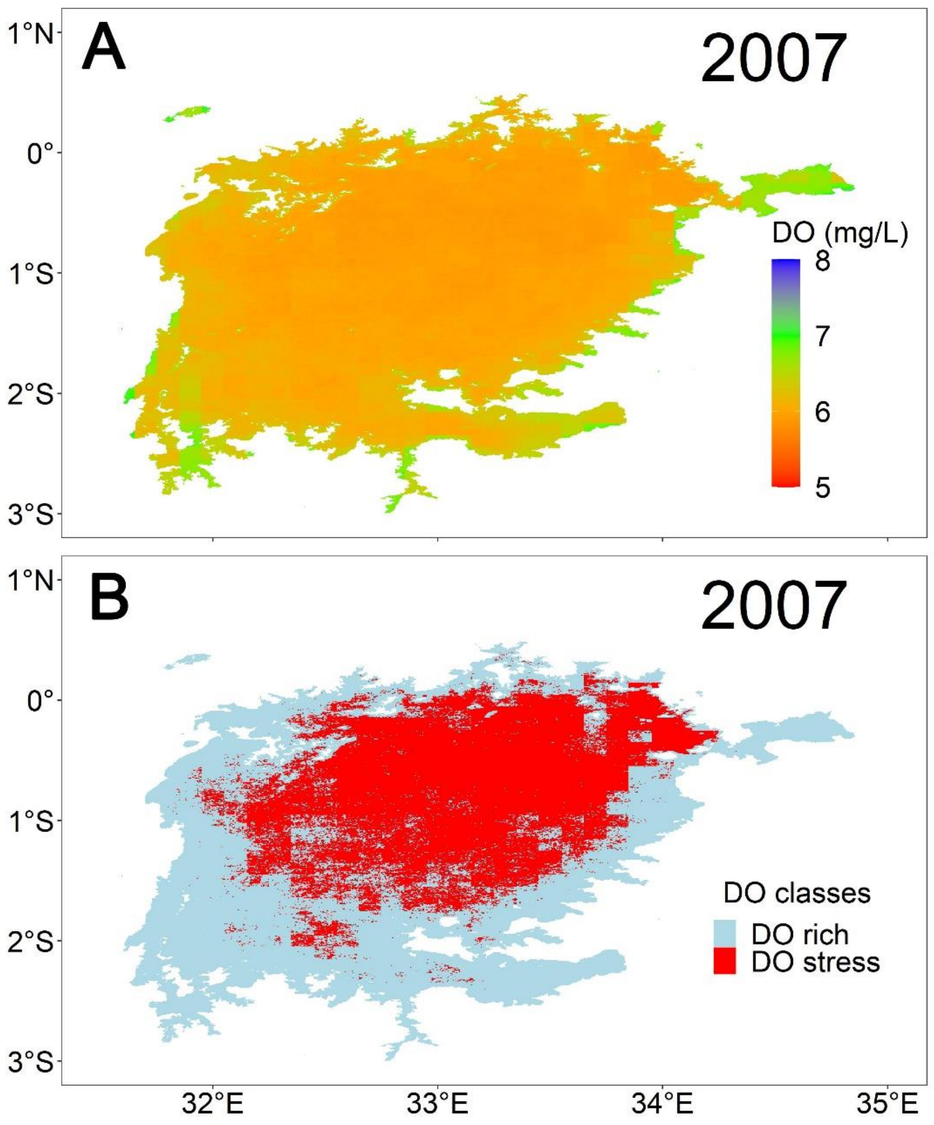

Fig. S16. **Dissolved oxygen concentrations in Lake Victoria.** (**A**) Surface oxygen levels in Lake Victoria during 2007. (**B**) Surface oxygen classes in Lake Victoria during 2007. Low-oxygen areas have expanded to 28,760 km^2, account for 43% of total surface area as a result of eutrophication and warming waters [90] (red, DO concentration ≤6 mg/L; blue, rich DO with concentration >6 mg/L).

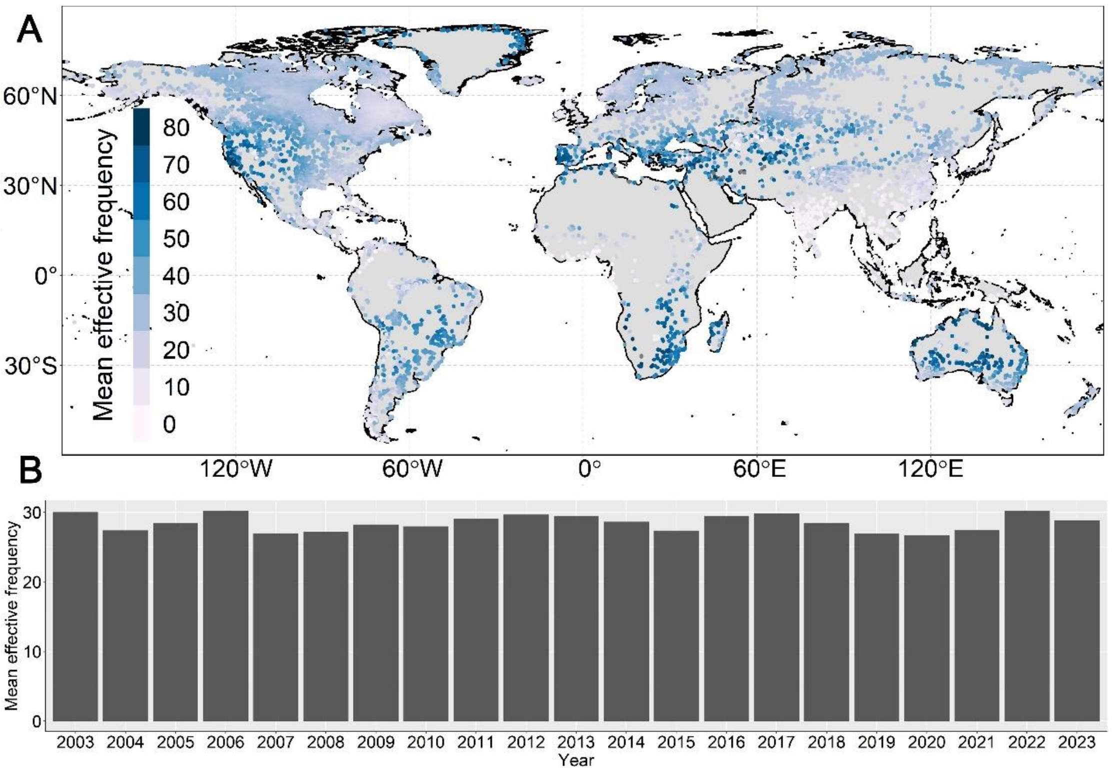

Fig. S17. **Spatial and temporal coverage of MODIS data over global lakes**. **(A)** Spatial mean coverage of MODIS data over global lakes during 2003-2023. **(B)** Time series of the mean coverage frequency of MODIS data over global lakes.

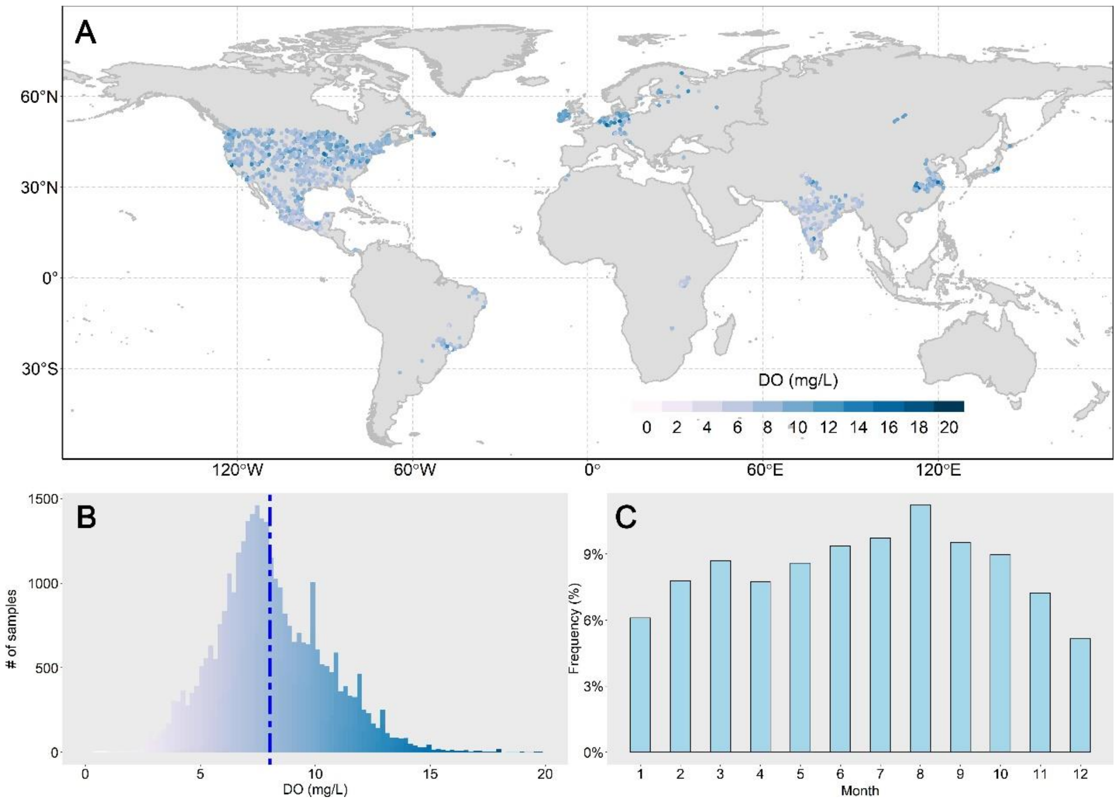

Fig. S18. Distribution of matchups between in situ measurements and concurrent MODIS observations. (**A**) Spatial distribution of matchups across global lakes. (**B**) Histogram statistics of the matchup count. (**C**) Seasonal distribution of matchups, illustrating the frequency across different seasons.

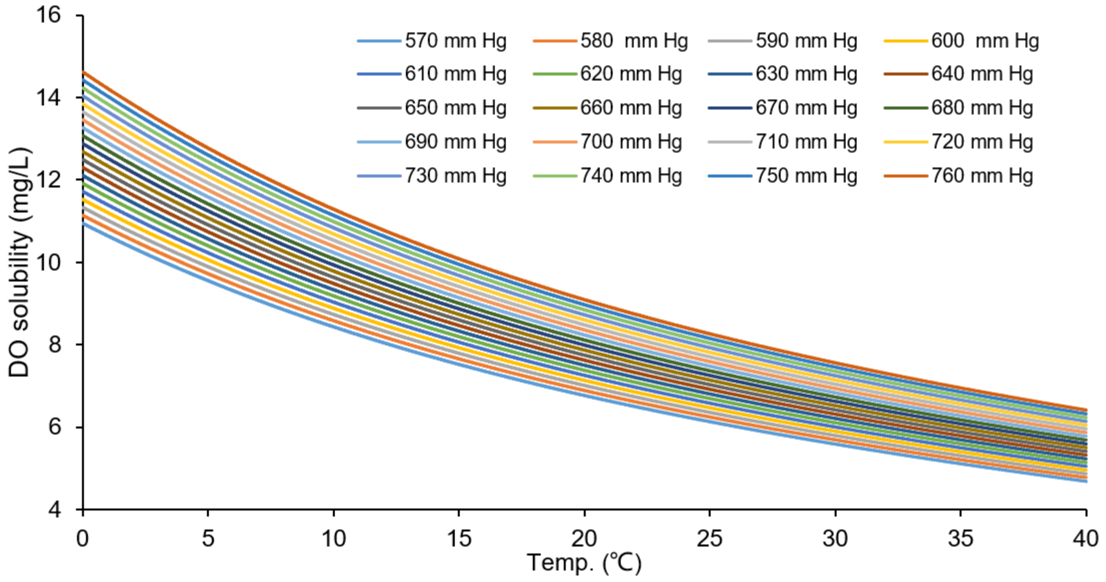

Fig. S19. Temperature and air pressure act as primary determinants of DO solubility. The solubility of oxygen exhibits a curved declining trend with increasing temperature. Under the same temperature conditions, higher atmospheric pressure leads to greater solubility.

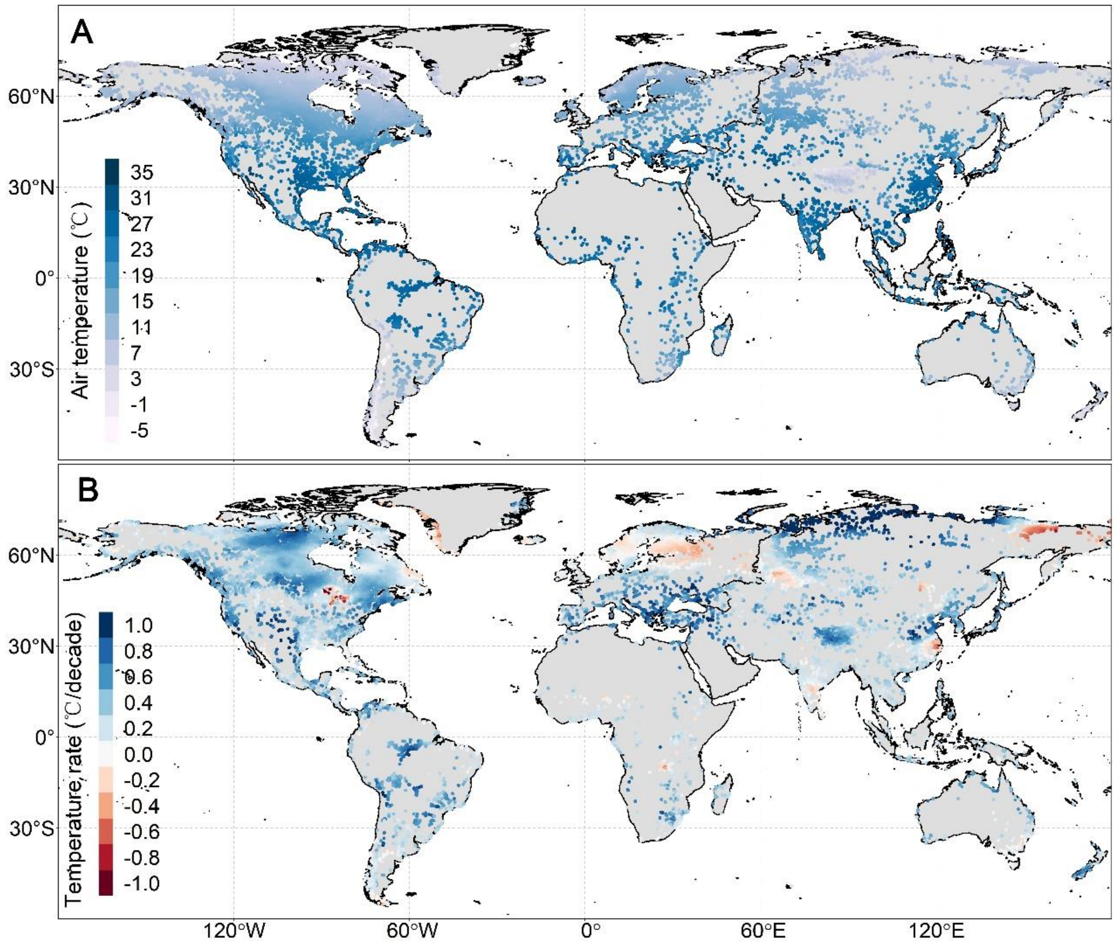

Fig. S20. Spatial and temporal variation of air temperature over global lakes from 2003 to 2023. (A) Spatial distribution of the mean air temperature. (**B**) Spatial distribution of the change rate.

Table S1. The statistics of model dataset across continents. The statistics were compiled based on the number of sampled lakes (No. of lakes) and sampling points (No. of observations) for each continent.

Continents	No. of lakes	No. of observations
North America	461	9514
Europe	98	7309
Asia	170	9475
Africa	5	3405
South America	20	3107

Table S2. Hyperparameters for BP, SVR, LSTM, XGBoost and RF models to estimate DO in this study. An optimal parameter list (optimal value) was derived by defining a hyperparameter grid (tuning range) and performing a grid search over 100 epochs for each model.

	Hyperparameter	Tuning range	Optimal value
BP	hidden_layer_sizes	(50,), (100,), (50, 50), (100, 50)	(100, 50)
	activation	relu, tanh, Sigmoid	'relu'
	alpha	1e-4, 1e-3, 1e-2, 1e-1	0.1
	learning_rate	0.01−1 with 0.01 interval	0.05
SVR	kernel	linear, rbf, sigmoid, poly	rbf
	epsilon	0.01−1.1 with 0.01 interval	0.08
	C	1-10 with 0.1 interval	8.5
	gamma	scale, auto	auto
	tol	1e-5, 1e-4, 1e-3, 1e-2, 1e-1	0.1
LSTM	units	100-200 with 10 intervals	120
	batch_size	2-20 with 2 intervals	12
	dropout_rate	0-1 with 0.1 intervals	0.1
	lr	0.001, 0.01, 0.1	0.01
XGBoost	learning_rate	0.01−0.3 with 0.01 interval	0.04
	n_estimators	10-1000 with 20 interval	120
	max_depth	1-10 with 1 interval	7
	min_child_weight	1-10 with 1 interval	2
	subsample	0.5-1 with 0.1 interval	0.7
	colsample_bytree	0.5-1 with 0.1 interval	0.7

RF	max_features	0-10 with 1 interval	1
	n_estimators	100-1000 with 100 intervals	500
	max_depth	10-100 with 10 intervals	50
	min_samples_split	2-20 with 2 intervals	4
	min_samples_leaf	0-10 with 1 interval	1
	max_leaf_nodes	0-2000 with 100 intervals	1000

REFERENCES AND NOTES

1. S. Schmidtko, L. Stramma, M. Visbeck, Decline in global oceanic oxygen content during the past five decades. *Nature* **542**, 335–339 (2017).

2. D. Gilbert, N. N. Rabalais, R. J. Díaz, J. Zhang, Evidence for greater oxygen decline rates in the coastal ocean than in the open ocean. *Biogeosciences.* **7**, 2283–2296 (2010).

3. W. Zhi, C. Klingler, J. Liu, L. Li, Widespread deoxygenation in warming rivers. *Nat. Clim. Chang.* **13**, 1105–1113 (2023).

4. D. Breitburg, L. A. Levin, A. Oschlies, M. Gregoire, F. P. Chavez, D. J. Conley, V. Garcon, D. Gilbert, D. Gutierrez, K. Isensee, G. S. Jacinto, K. E. Limburg, I. Montes, S. W. A. Naqvi, G. C. Pitcher, N. N. Rabalais, M. R. Roman, K. A. Rose, B. A. Seibel, M. Telszewski, M. Yasuhara, J. Zhang, Declining oxygen in the global ocean and coastal waters. *Science* **359**, eaam7240 (2018).

5. S. F. Jane, G. J. A. Hansen, B. M. Kraemer, P. R. Leavitt, J. L. Mincer, R. L. North, R. M. Pilla, J. T. Stetler, C. E. Williamson, R. I. Woolway, L. Arvola, S. Chandra, C. L. DeGasperi, L. Diemer, J. Dunalska, O. Erina, G. Flaim, H. P. Grossart, K. D. Hambright, C. Hein, J. Hejzlar, L. L. Janus, J. P. Jenny, J. R. Jones, L. B. Knoll, B. Leoni, E. Mackay, S. S. Matsuzaki, C. McBride, D. C. Muller-Navarra, A. M. Paterson, D. Pierson, M. Rogora, J. A. Rusak, S. Sadro, E. Saulnier-Talbot, M. Schmid, R. Sommaruga, W. Thiery, P. Verburg, K. C. Weathers, G. A. Weyhenmeyer, K. Yokota, K. C. Rose, Widespread deoxygenation of temperate lakes. *Nature* **594**, 66–70 (2021).

6. K. C. Rose, E. M. Ferrer, S. R. Carpenter, S. A. Crowe, S. C. Donelan, V. C. Garcon, M. Gregoire, S. F. Jane, P. R. Leavitt, L. A. Levin, A. Oschlies, D. Breitburg, Aquatic deoxygenation as a planetary boundary and key regulator of Earth system stability. *Nat. Ecol. Evol.* **8**, 1400–1406 (2024).

7. R. J. Diaz, R. Rosenberg, Spreading dead zones and consequences for marine ecosystems. *Science* **321**, 926–929 (2008).

8. R. E. Keeling, A. Kortzinger, N. Gruber, Ocean deoxygenation in a warming world. *Ann. Rev. Mar. Sci.* **2**, 199–229 (2010).

9. L. Stramma, E. D. Prince, S. Schmidtko, J. Luo, J. P. Hoolihan, M. Visbeck, D. W. R. Wallace, P. Brandt, A. Körtzinger, Expansion of oxygen minimum zones may reduce available habitat for tropical pelagic fishes. *Nat. Clim. Chang.* **2**, 33–37 (2011).

10. R. Vaquer-Sunyer, C. M. Duarte, Thresholds of hypoxia for marine biodiversity. *Proc. Natl. Acad. Sci. U.S.A.* **105**, 15452–15457 (2008).

11. R. I. Woolway, C. J. Merchant, Worldwide alteration of lake mixing regimes in response to climate change. *Nat. Geosci.* **12**, 271–276 (2019).

12. R. F. Keeling, H. E. Garcia, The change in oceanic O_2 inventory associated with recent global warming. *Proc. Natl. Acad. Sci. U.S.A.* **99**, 7848–7853 (2002).

13. J. R. Blaszczak, L. E. Koenig, F. H. Mejia, L. Gómez-Gener, C. L. Dutton, A. M. Carter, N. B. Grimm, J. W. Harvey, A. M. Helton, M. J. Cohen, Extent, patterns, and drivers of hypoxia in the world's streams and rivers. *Limnol. Oceanogr. Lett.* **8**, 453–463 (2022).

14. L. L. Yuan, A. I. Pollard, Classifying lakes to quantify relationships between epilimnetic chlorophyll a and hypoxia. *Environ. Manag.* **55**, 578–587 (2015).

15. N. N. Rabalais, R. J. D'ıaz, L. A. Levin, R. E. Turner, D. Gilbert, J. Zhang, Dynamics and distribution of natural and human-caused hypoxia. *Biogeosciences* **7**, 585–619 (2010).

16. J. Karstensen, B. Fiedler, F. Schütte, P. Brandt, A. Körtzinger, G. Fischer, R. Zantopp, J. Hahn, M. Visbeck, D. Wallace, Open ocean dead zones in the tropical North Atlantic Ocean. *Biogeosciences* **12**, 2597–2605 (2015).

17. S. Kosten, V. L. M. Huszar, E. Bécares, L. S. Costa, E. van Donk, L. A. Hansson, E. Jeppesen, C. Kruk, G. Lacerot, N. Mazzeo, L. De Meester, B. Moss, M. Lürling, T. Nõges, S. Romo, M. Scheffer, Warmer climates boost cyanobacterial dominance in shallow lakes. *Glob. Chang. Biol.* **18**, 118–126 (2011).

18. A. M. Carter, J. R. Blaszczak, J. B. Heffernan, E. S. Bernhardt, Hypoxia dynamics and spatial distribution in a low gradient river. *Limnol. Oceanogr.* **66**, 2251–2265 (2021).

19. J. P. Jenny, P. Francus, A. Normandeau, F. Lapointe, M. E. Perga, A. Ojala, A. Schimmelmann, B. Zolitschka, Global spread of hypoxia in freshwater ecosystems during the last three centuries is caused by rising local human pressure. *Glob. Chang. Biol.* **22**, 1481–1489 (2016).

20. R. P. North, R. L. North, D. M. Livingstone, O. Koster, R. Kipfer, Long-term changes in hypoxia and soluble reactive phosphorus in the hypolimnion of a large temperate lake: Consequences of a climate regime shift. *Glob. Chang. Biol.* **20**, 811–823 (2014).

21. W. J. Wang, K. Shi, X. W. Wang, Y. L. Zhang, B. Q. Qin, Y. B. Zhang, R. I. Woolway, The impact of extreme heat on lake warming in China. *Nat. Commun.* **15**, 70 (2024).

22. R. I. Woolway, E. Jennings, T. Shatwell, M. Golub, D. C. Pierson, S. C. Maberly, Lake heatwaves under climate change. *Nature* **589**, 402–407 (2021).

23. R. Shinohara, S. I. S. Matsuzaki, M. Watanabe, M. Nakagawa, H. Yoshida, A. Kohzu, Heat waves can cause hypoxia in shallow lakes. *Geophys. Res. Lett.* **50**, e2023GL102967 (2023).

24. T. Jankowski, D. M. Livingstone, H. Bührer, R. Forster, P. Niederhauser, Consequences of the 2003 European heat wave for lake temperature profiles, thermal stability, and hypolimnetic oxygen depletion: Implications for a warmer world. *Limnol. Oceanogr.* **51**, 815–819 (2006).

25. C. Hu, A novel ocean color index to detect floating algae in the global oceans. *Remote Sens. Environ.* **113**, 2118–2129 (2009).

26. S. Wang, J. Li, Q. Shen, B. Zhang, F. Zhang, Z. Lu, MODIS-based radiometric color extraction and classification of inland water with the Forel-Ule scale: A case study of Lake Taihu. *IEEE J. Sel. Top. Appl. Earth Obs. Remote Sens.* **8**, 907–918 (2015).

27. X. Hou, L. Feng, Y. Dai, C. Hu, L. Gibson, J. Tang, Z. Lee, Y. Wang, X. Cai, J. Liu, Y. Zheng, C. Zheng, Global mapping reveals increase in lacustrine algal blooms over the past decade. *Nat. Geosci.* **15**, 130–134 (2022).

28. S. L. Wang, J. S. Li, B. Zhang, E. Spyrakos, A. N. Tyler, Q. Shen, F. F. Zhang, T. Kuster, M. K. Lehmann, Y. H. Wu, D. L. Peng, Trophic state assessment of global inland waters using a MODIS-derived Forel-Ule index. *Remote Sens. Environ.* **217**, 444–460 (2018).

29. L. B. Chi, X. X. Song, Y. Q. Yuan, W. T. Wang, X. H. Cao, Z. X. Wu, Z. M. Yu, Main factors dominating the development, formation and dissipation of hypoxia off the Changjiang Estuary (CE) and its adjacent waters, China. *Environ. Pollut.* **265**, 115066 (2020).

30. J. Rosenfeld, R. Lee, Thresholds for reduction in fish growth and consumption due to hypoxia: Implications for water quality guidelines to protect aquatic life. *Environ. Manag.* **70**, 431–447 (2022).

31. S. C. Chapra, B. Boehlert, C. Fant, V. J. Bierman Jr., J. Henderson, D. Mills, D. M. L. Mas, L. Rennels, L. Jantarasami, J. Martinich, K. M. Strzepek, H. W. Paerl, Climate change impacts on harmful algal blooms in U.S. Freshwaters: A screening-level assessment. *Environ. Sci. Technol.* **51**, 8933–8943 (2017).

32. J. C. Ho, A. M. Michalak, N. Pahlevan, Widespread global increase in intense lake phytoplankton blooms since the 1980s. *Nature* **574**, 667–670 (2019).

33. P. Shao, X. Zeng, X. Zeng, Differences in carbon cycle and temperature projections from emission- and concentration-driven earth system model simulations. *Earth Syst. Dyn. Discuss.* **5**, 991–1012 (2014).

34. P. D. Jones, M. New, D. E. Parker, S. Martin, I. G. Rigor, Surface air temperature and its changes over the past 150 years. *Rev. Geophys.* **37**, 173–199 (1999).

35. S. R. Carpenter, E. H. Stanley, M. J. Vander Zanden, State of the World's freshwater ecosystems: Physical, chemical, and biological changes. *Annu. Rev. Env. Resour.* **36**, 75–99 (2011).

36. H. W. Paerl, J. Huisman, Blooms like it hot. *Science* **320**, 57–58 (2008).

37. R. D. Robarts, T. Zohary, Temperature effects on photosynthetic capacity, respiration, and growth rates of bloom-forming cyanobacteria. *N. Z. J. Mar. Freshw. Res.* **21**, 391–399 (1987).

38. E. Martí, J. L. Riera, F. Sabater, "Effects of wastewater treatment plants on stream nutrient dynamics under water scarcity conditions" in *Water Scarcity in the Mediterranean: Perspectives Under Global Change*, S. Sabater, D. Barceló, Eds. (Springer Berlin Heidelberg, 2010), pp. 173 195.

39. X. Zhang, E. A. Davidson, D. L. Mauzerall, T. D. Searchinger, P. Dumas, Y. Shen, Managing nitrogen for sustainable development. *Nature* **528**, 51–59 (2015).

40. M. W. Doyle, E. H. Stanley, D. G. Havlick, M. J. Kaiser, G. Steinbach, W. L. Graf, G. E. Galloway, J. A. Riggsbee, Aging infrastructure and ecosystem restoration. *Science* **319**, 286–287 (2008).

41. W. Zou, H. Xu, G. Zhu, M. Zhu, C. Guo, M. Xiao, Y. Zhang, B. Qin, Why do algal blooms intensify under reduced nitrogen and fluctuating phosphorus conditions: The underappreciated role of non-algal light attenuation. *Limnol. Oceanogr.* **68**, 2274–2287 (2023).

42. T. Posch, O. Köster, M. M. Salcher, J. Pernthaler, Harmful filamentous cyanobacteria favoured by reduced water turnover with lake warming. *Nat. Clim. Chang.* **2**, 809–813 (2012).

43. IPCC, Climate Change 2021, *The Physical Science Basis*, V. Masson-Delmotte, A. Zhai, Pirani, S. L. Connors, C. Péan, S. Berger, N. Caud, Y. Chen, L. Goldfarb, M. I. Gomis, M. Huang, K. Leitzell, E. Lonnoy, J. B. R. Matthews, T. K. Maycock, T. Waterfield, O. Yelekçi, R. Yu, B. Zhou, Eds. (Cambridge Univ. Press, 2021). pp. 2391.

44. L. Deirmendjian, J. P. Descy, C. Morana, W. Okello, M. P. Stoyneva-Gärtner, S. Bouillon, A. V. Borges, Limnological changes in Lake Victoria since the mid-20th century. *Freshw. Biol.* **66**, 1630–1647 (2021).

45. G. W. Ngupula, C. N. Ezekiel, I. A. Kimirei, E. Mboni, B. B. Kashindye, Physical and chemical characteristics of the Tanzanian inshore and offshore waters of Lake Victoria in 2005–2008. *Afr. J. Aquat. Sci.* **37**, 339–345 (2012).

46. D. Breitburg, Effects of hypoxia, and the balance between hypoxia and enrichment, on coastal fishes and fisheries. *Estuaries* **25**, 767–781 (2002).

47. N. Gruber, *The Ocean Carbon Cycle and Climate*, M. Follows, T. Oguz, Eds. (Springer Netherlands, 2004), pp. 97–148.

48. D. L. Arévalo-Martínez, A. Kock, C. R. Löscher, R. A. Schmitz, H. W. Bange, Massive nitrous oxide emissions from the tropical South Pacific Ocean. *Nat. Geosci.* **8**, 530–533 (2015).

49. D. A. Hutchins, D. G. Capone, The marine nitrogen cycle: New developments and global change. *Nat. Rev. Microbiol.* **20**, 401–414 (2022).

50. L. Bohlen, A. W. Dale, K. Wallmann, Simple transfer functions for calculating benthic fixed nitrogen losses and C:N:P regeneration ratios in global biogeochemical models. *Global Biogeochem. Cycles* **26**, GB3029 (2012).

51. E. Ingall, R. Jahnke, Evidence for enhanced phosphorus regeneration from marine sediments overlain by oxygen depleted waters. *Geochim. Cosmochim. Acta* **58**, 2571–2575 (1994).

52. F. Scholz, J. McManus, A. C. Mix, C. Hensen, R. R. Schneider, The impact of ocean deoxygenation on iron release from continental margin sediments. *Nat. Geosci.* **7**, 433–437 (2014).

53. D. J. Conley, J. Carstensen, R. Vaquer-Sunyer, C. M. Duarte, Ecosystem thresholds with hypoxia. *Hydrobiologia* **629**, 21–29 (2009).

54. R. G. Wetzel, *Limnology: Lake and River Ecosystems* (Elsevier Science, 2001).

55. X. J. Hou, L. Feng, H. T. Duan, X. L. Chen, D. Y. Sun, K. Shi, Fifteen-year monitoring of the turbidity dynamics in large lakes and reservoirs in the middle and lower basin of the Yangtze River, China. *Remote Sens. Environ.* **190**, 107–121 (2017).

56. N.-B. Chang, Z. Xuan, Y. J. Yang, Exploring spatiotemporal patterns of phosphorus concentrations in a coastal bay with MODIS images and machine learning models. *Remote Sens. Environ.* **134**, 100–110 (2013).

57. S. Constantin, D. Doxaran, Ș. Constantinescu, Estimation of water turbidity and analysis of its spatio-temporal variability in the Danube River plume (Black Sea) using MODIS satellite data. *Cont. Shelf Res.* **112**, 14–30 (2016).

58. B. Khazaei, L. K. Read, M. Casali, K. M. Sampson, D. N. Yates, GLOBathy, the global lakes bathymetry dataset. *Sci. Data* **9**, 36 (2022).

59. IOCCG. Update of the Quasi-Analytical Algorithm (QAA_v6). IOCCG (2014); http://ioccg.org/groups/Software_OCA/QAA_v6_2014209.pdf.

60. L. I. W. McKinna, P. J. Werdell, Approach for identifying optically shallow pixels when processing ocean-color imagery. *Opt. Express* **26**, A915–A928 (2018).

61. N. Gorelick, M. Hancher, M. Dixon, S. Ilyushchenko, D. Thau, R. Moore, Google Earth Engine: Planetary-scale geospatial analysis for everyone. *Remote Sens. Environ.* **202**, 18–27 (2017).

62. Z. Cao, C. Hu, R. Ma, H. Duan, M. Liu, S. Loiselle, K. Song, M. Shen, D. Liu, K. Xue, MODIS observations reveal decrease in lake suspended particulate matter across China over the past two decades. *Remote Sens. Environ.* **295**, 113724 (2023).

63. M. L. Messager, B. Lehner, G. Grill, I. Nedeva, O. Schmitt, Estimating the volume and age of water stored in global lakes using a geo-statistical approach. *Nat. Commun.* **7**, 13603 (2016).

64. G. L. Feyisa, H. Meilby, R. Fensholt, S. R. Proud, Automated Water Extraction Index: A new technique for surface water mapping using Landsat imagery. *Remote Sens. Environ.* **140**, 23–35 (2014).

65. J. Muñoz-Sabater, E. Dutra, A. Agustí-Panareda, C. Albergel, G. Arduini, G. Balsamo, S. Boussetta, M. Choulga, S. Harrigan, H. Hersbach, B. Martens, D. G. Miralles, M. Piles, N. J.

Rodríguez-Fernández, E. Zsoter, C. Buontempo, J. N. Thépaut, ERA5-Land: a state-of-the-art global reanalysis dataset for land applications. *Earth Syst. Sci. Data* **13**, 4349-4383 (2021).

66. M. A. Warren, S. G. H. Simis, V. Martinez-Vicente, K. Poser, M. Bresciani, K. Alikas, E. Spyrakos, C. Giardino, A. Ansper, Assessment of atmospheric correction algorithms for the Sentinel-2A MultiSpectral Imager over coastal and inland waters. *Remote Sens. Environ.* **225**, 267–289 (2019).

67. W. Zhi, W. Ouyang, C. Shen, L. Li, Temperature outweighs light and flow as the predominant driver of dissolved oxygen in US rivers. *Nat. Water* **1**, 249–260 (2023).

68. M. Li, Y. J. Lee, J. M. Testa, Y. Li, W. Ni, W. M. Kemp, D. M. Di Toro, What drives interannual variability of hypoxia in Chesapeake Bay: Climate forcing versus nutrient loading? *Geophys. Res. Lett.* **43**, 2127–2134 (2016).

69. M. E. Scully, The importance of climate variability to wind-driven modulation of hypoxia in Chesapeake Bay. *J. Phys. Oceanogr.* **40**, 1435–1440 (2010).

70. J. S. Lefcheck, piecewiseSEM: Piecewise structural equation modelling in r for ecology, evolution, and systematics. *Methods Ecol. Evol.* **7**, 573–579 (2016).

71. S. V. Balasubramanian, N. Pahlevan, B. Smith, C. Binding, J. Schalles, H. Loisel, D. Gurlin, S. Greb, K. Alikas, M. Randla, M. Bunkei, W. Moses, H. Nguyễn, M. K. Lehmann, D. O'Donnell, M. Ondrusek, T.-H. Han, C. G. Fichot, T. Moore, E. Boss, Robust algorithm for estimating total suspended solids (TSS) in inland and nearshore coastal waters. *Remote Sens. Environ.* **246**, 111768 (2020).

72. F. Giannini, B. P. V. Hunt, D. Jacoby, M. Costa, Performance of OLCI Sentinel-3A satellite in the Northeast Pacific coastal waters. *Remote Sens. Environ.* **256**, 112317 (2021).

73. M. Shen, H. T. Duan, Z. G. Cao, K. Xue, T. C. Qi, J. G. Ma, D. Liu, K. S. Song, C. L. Huang, X. Y. Song, Sentinel-3 OLCI observations of water clarity in large lakes in eastern China: Implications for SDG 6.3.2 evaluation. *Remote Sens. Environ.* **247**, 111950 (2020).

74. H. Z. Liu, Q. Q. Li, Y. Bai, C. Yang, J. J. Wang, Q. M. Zhou, S. B. Hu, T. Z. Shi, X. M. Liao, G. F. Wu, Improving satellite retrieval of oceanic particulate organic carbon concentrations using machine learning methods. *Remote Sens. Environ.* **256**, 112316 (2021).

75. Y. B. Zhang, K. Shi, X. Sun, Y. L. Zhang, N. Li, W. J. Wang, Y. Q. Zhou, W. Zhi, M. L. Liu, Y. Li, G. W. Zhu, B. Q. Qin, E. Jeppesen, J. Zhou, H. Y. Li, Improving remote sensing estimation of Secchi disk depth for global lakes and reservoirs using machine learning methods. *GISci. Remote Sens.* **59**, 1367–1383 (2022).

76. S. Hochreiter, J. Schmidhuber, Long short-term memory. *Neural Comput.* **9**, 1735–1780 (1997).

77. J. Du, J. S. Kimball, C. Duguay, Y. Kim, J. D. Watts, Satellite microwave assessment of Northern Hemisphere lake ice phenology from 2002 to 2015. *Cryosphere* **11**, 47–63 (2017).

78. D. C. Pierson, G. A. Weyhenmeyer, L. Arvola, B. Benson, T. Blenckner, T. Kratz, D. M. Livingstone, H. Markensten, G. Marzec, K. Pettersson, K. Weathers, An automated method to monitor lake ice phenology. *Limnol. Oceanogr. Methods* **9**, 74–83 (2011).

79. R. Hugonnet, R. McNabb, E. Berthier, B. Menounos, C. Nuth, L. Girod, D. Farinotti, M. Huss, I. Dussaillant, F. Brun, A. Kaab, Accelerated global glacier mass loss in the early twenty-first century. *Nature* **592**, 726–731 (2021).

80. P. Virtanen, R. Gommers, T. E. Oliphant, M. Haberland, T. Reddy, D. Cournapeau, E. Burovski, P. Peterson, W. Weckesser, J. Bright, S. J. van der Walt, M. Brett, J. Wilson, K. J. Millman, N. Mayorov, A. R. J. Nelson, E. Jones, R. Kern, E. Larson, C. J. Carey, İ. Polat, Y. Feng, E. W. Moore, J. VanderPlas, D. Laxalde, J. Perktold, R. Cimrman, I. Henriksen, E. A. Quintero, C. R. Harris, A. M. Archibald, A. H. Ribeiro, F. Pedregosa, P. van Mulbregt, A. Vijaykumar, A. P. Bardelli, A. Rothberg, A. Hilboll, A. Kloeckner, A. Scopatz, A. Lee, A. Rokem, C. N. Woods, C. Fulton, C. Masson, C. Häggström, C. Fitzgerald, D. A. Nicholson, D. R. Hagen, D. V. Pasechnik, E. Olivetti, E. Martin, E. Wieser, F. Silva, F. Lenders, F. Wilhelm, G. Young, G. A. Price, G.-L. Ingold, G. E. Allen, G. R. Lee, H. Audren, I. Probst, J. P. Dietrich, J. Silterra, J. T. Webber, J. Slavič, J. Nothman, J. Buchner, J. Kulick, J. L. Schönberger, J. V. de Miranda Cardoso, J. Reimer, J. Harrington, J. L. C. Rodríguez, J.

Nunez-Iglesias, J. Kuczynski, K. Tritz, M. Thoma, M. Newville, M. Kümmerer, M. Bolingbroke, M. Tartre, M. Pak, N. J. Smith, N. Nowaczyk, N. Shebanov, O. Pavlyk, P. A. Brodtkorb, P. Lee, R. T. McGibbon, R. Feldbauer, S. Lewis, S. Tygier, S. Sievert, S. Vigna, S. Peterson, S. More, T. Pudlik, T. Oshima, T. J. Pingel, T. P. Robitaille, T. Spura, T. R. Jones, T. Cera, T. Leslie, T. Zito, T. Krauss, U. Upadhyay, Y. O. Halchenko, Y. Vázquez-Baeza, C. SciPy, SciPy 1.0: Fundamental algorithms for scientific computing in Python. *Nat. Methods* **17**, 261–272 (2020).

81. H. B. Mann, Non-parametric tests against trend. *Econometrica* **13**, 163–171 (1945).

82. M. G. Kendall, *Rank Correlation Measures* (Charles Griffin, ed. 4, 1975.

83. C. Kuhn, D. Butman, Declining greenness in Arctic-boreal lakes. *Proc. Natl. Acad. Sci. U.S.A.* **118**, e2021219118 (2021).

84. L. V. Alexander, S. E. Perkins, On the measurement of heat waves. *J. Climate* **26**, 4500–4517 (2013).

85. A. J. Hobday, L. V. Alexander, S. E. Perkins, D. A. Smale, S. C. Straub, E. C. J. Oliver, J. A. Benthuysen, M. T. Burrows, M. G. Donat, M. Feng, N. J. Holbrook, P. J. Moore, H. A. Scannell, A. Sen Gupta, T. Wernberg, A hierarchical approach to defining marine heatwaves. *Prog. Oceanogr.* **141**, 227–238 (2016).

86. D. E. Rumelhart, G. E. Hinton, R. J. Williams, Learning representations by back-propagating errors. *Nature* **323**, 533–536 (1986).

87. O. A. Montesinos López, A. Montesinos López, J. Crossa, "Support vector machines and support vector regression" in *Multivariate Statistical Machine Learning Methods for Genomic Prediction*, O. A. Montesinos López, A. Montesinos López, J. Crossa, Eds. (Springer International Publishing, 2022), pp. 337–378.

88. L. Breiman, Random forests. *Mach. Learn.* **45**, 5–32 (2001).

89. T. Q. Chen, T. He, M. Benesty, V. Khotilovich, Y. Tang, H. Cho, Xgboost: Extreme gradient boosting. *R package version* 0.4-2 1, 1–4 (2015).

90. J. Njiru, M. van der Knaap, R. Kundu, C. Nyamweya, Lake Victoria fisheries: Outlook and management. *Lakes Reservoirs Res. Manag.* **23**, 152–162 (2018).
